# NDRG1‐Driven Lactate Accumulation Promotes Lung Adenocarcinoma Progression Through the Induction of an Immunosuppressive Microenvironment

**DOI:** 10.1002/advs.202501238

**Published:** 2025-06-20

**Authors:** Gujie Wu, Hongxia Cheng, Jiacheng Yin, Yuansheng Zheng, Haochun Shi, Binyang Pan, Ming Li, Mengnan Zhao, Jiaqi Liang, Yunyi Bian, Guangyao Shan, Guoshu Bi, Weigang Guo, Lin Wang, Yiwei Huang

**Affiliations:** ^1^ Department of Thoracic Surgery Zhongshan Hospital Fudan University Shanghai 200032 China; ^2^ Cancer Research Center Institute of Biomedical Science Fudan University Shanghai 200032 China; ^3^ Department of Radiation Oncology Shanghai Pulmonary Hospital Tongji University School of Medicine Shanghai 200032 China

**Keywords:** immunotherapy, lactylation, lung adenocarcinoma, NDRG1, tumor microenvironment

## Abstract

Lung adenocarcinoma (LUAD) is a leading cause of cancer‐related mortality, with the tumor microenvironment (TME) playing a critical role in its progression. Metabolic reprogramming, particularly lactate accumulation, drives immune suppression within the TME. Utilizing single‐cell RNA sequencing (scRNA‐seq) of 30 LUAD samples, genome‐wide association studies (GWAS) involving 29,863 patients and 55,586 controls, and clinical data from 220 LUAD patients, we identified N‐Myc downstream‐regulated gene 1 (NDRG1) as a key pathogenic gene in LUAD, strongly associated with tumor progression and poor prognosis. Mechanistic studies revealed that NDRG1 stabilizes lactate dehydrogenase A (LDHA) by inhibiting its ubiquitination, thereby enhancing glycolysis and promoting lactate accumulation. This process fosters immune suppression by inducing M2 macrophage polarization, impairing CD8^+^ T cell function, and upregulating immunosuppressive genes. Furthermore, histone H3K18 lactylation in macrophages exacerbates this immunosuppressive state. Clinically, elevated NDRG1 expression correlates with increased PD‐L1 levels, a higher abundance of immunosuppressive macrophages, and reduced CD8^+^ T cell infiltration, contributing to immunotherapy resistance. Conversely, low NDRG1 expression is associated with enhanced CD8^+^ T cell infiltration and improved therapeutic outcomes. Preclinical studies demonstrated targeting NDRG1 suppresses tumor growth, alleviates immune suppression, and boosts anti‐PD‐L1 efficacy. These findings establish NDRG1 as a critical LUAD regulator and a promising immunotherapy target.

## Graphical abstract

NDRG1 drives lactate accumulation and histone lactylation in lung adenocarcinoma, promoting immunosuppression via M2 macrophage polarization and CD8⁺ T cell inhibition. High NDRG1 expression correlates with poor prognosis, an immunosuppressive microenvironment, and immunotherapy resistance. Targeting NDRG1 reverses lactate‐driven metabolic reprogramming and epigenetic modifications, restoring immune activity and enhancing anti‐PD‐L1 therapy efficacy.

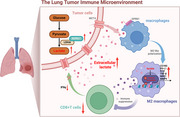

## 1 Introduction

Lung cancer is one of the most common malignancies worldwide and the leading cause of cancer‐related mortality.^[^ ^1^ ^]^ Among lung cancers, lung adenocarcinoma is the predominant subtype of non‐small cell lung cancer (NSCLC), and significant advancements in its treatment strategies have been made in recent years.^[^ ^2^ ^]^ Despite the effectiveness of traditional therapies such as surgery, radiotherapy, and chemotherapy, substantial challenges remain in advanced or metastatic lung adenocarcinoma.^[^ ^3^ ^]^ With the growing understanding of the tumor microenvironment (TME) and the advent of immunotherapy, the therapeutic outlook for lung adenocarcinoma has fundamentally improved.^[^ ^4^ ^]^ Immunotherapy, by activating the patient's immune system to recognize and eliminate cancer cells, has shown significant potential in improving patient survival rates.^[^ ^5^ ^]^ Additionally, interdisciplinary research is crucial for comprehensively understanding the complexity and immune evasion mechanisms of lung adenocarcinoma, driving the development of precision treatment strategies, and identifying new immunotherapeutic targets to improve patient outcomes.^[^ ^6^ ^]^

Lactate metabolism and its impact on the tumor microenvironment have become major research focuses in recent years.^[^ ^7^, ^8^, ^9^ ^]^ Cancer cells commonly exhibit enhanced glycolysis (known as the Warburg effect), leading to the production of large amounts of lactate.^[^ ^10^ ^]^ This not only alters the metabolic landscape of the tumor microenvironment but also acts as a critical signal that promotes tumor cell growth and survival while exerting profound inhibitory effects on immune cell function.^[^ ^11^, ^12^, ^13^, ^14^, ^15^ ^]^ This has highlighted the potential of targeting lactate metabolism as a novel cancer therapeutic strategy; by modulating lactate production and function, it may be possible to reverse the acidic tumor microenvironment, restore immune cell functionality, and enhance antitumor immune responses.^[^ ^16^ ^]^ In this context, N‐Myc downstream‐regulated gene 1 (NDRG1) has emerged as a key regulatory factor in the tumor microenvironment. Studies have shown that NDRG1 not only regulates cellular stress responses, growth, and apoptosis but also plays a crucial role in metabolism processes.^[^ ^17^, ^18^ ^]^ Understanding the specific role of NDRG1 in these metabolic processes is of great importance for developing new cancer therapeutic strategies.

This study aims to explore the intricate relationship between metabolic reprogramming and immune regulation within the tumor microenvironment, and how these factors collectively influence tumor progression and immune evasion. To this end, we initially employed single‐cell RNA sequencing (scRNA‐seq) to classify and analyze different cell types within lung adenocarcinoma (LUAD) samples. Subsequently, we utilized extensive genetic data from clinical patients, integrated with genome‐wide association studies (GWAS), to identify NDRG1 as a key gene and validated its causal relationship with tumor progression through Mendelian randomization (MR). We further investigated the role of NDRG1 in metabolic reprogramming and examined how these metabolic alterations affect immune cell behavior. In vitro and in vivo experiments were conducted to validate the metabolic and immunomodulatory roles of NDRG1. This comprehensive approach lays the groundwork for designing novel immunotherapeutic strategies. By integrating clinical and molecular research, we aim to provide new perspectives for the treatment of LUAD, explore new immunotherapeutic targets, and offer essential scientific evidence for developing new treatments and improving patient outcomes.

## 2 Result

### 2.1 NDRG1 is Significantly Upregulated in LUAD and Indicates a Poor Prognosis

**Figure 1:**
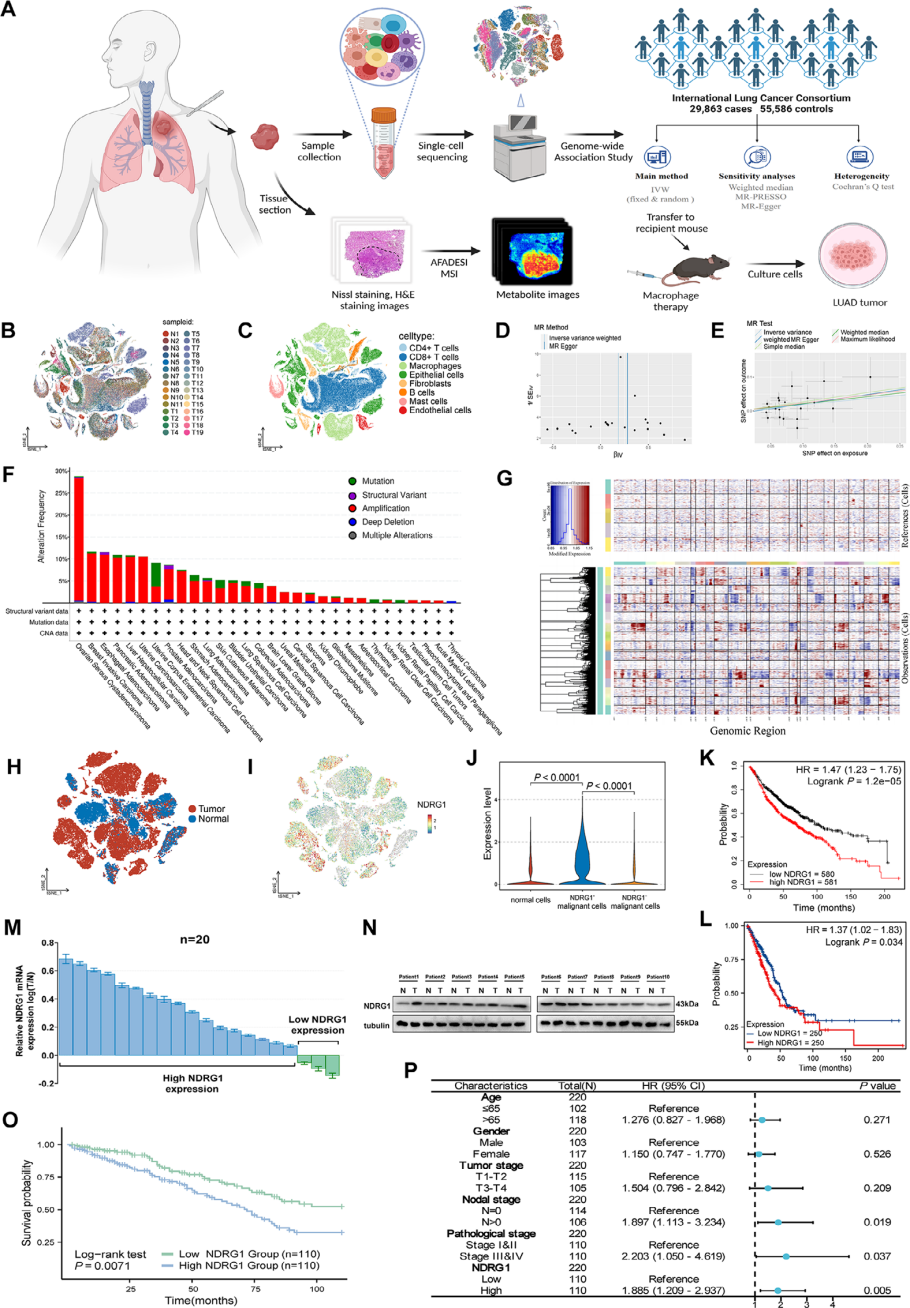
NDRG1 is significantly upregulated in LUAD and indicates poor prognosis. A) Flowchart illustrating the study design. B) t‐SNE plot showing the origin and characteristics of single cells. C) t‐SNE plot showing single cells categorized by different cell types. D) Funnel plot for heterogeneity testing between NDRG1 and outcome factors. E) Scatter plot for pleiotropy testing between NDRG1 and outcome factors. F) Genomic alterations of NDRG1 across various human cancer types from TCGA data, including mutations, amplifications, and deep deletions, shown as percentage frequencies. G) CNV analysis heatmap in malignant and non‐malignant cells. H) t‐SNE plot of clusters of normal and malignant cells. I) t‐SNE plot showing NDRG1 expression in clusters of normal and malignant cells. J) Violin plot comparing the expression levels in normal, NDRG1^+^, and NDRG1^‐^ malignant cells. K) Prognostic analysis of NDRG1 from the KM database. L) Prognostic analysis of NDRG1 from TCGA database. M) Quantitative analysis of NDRG1 mRNA levels in 20 pairs of matched LUAD and adjacent non‐tumor tissues using qRT‐PCR. N) Western blot analysis of NDRG1 in cancer and adjacent non‐tumor samples (n=3). O) Kaplan‐Meier survival curves comparing patients with high and low NDRG1 expression. P) Multivariate Cox regression analysis of clinical factors and prognosis in lung adenocarcinoma patients. Differences were considered statistically significant at *p* < 0.05.

In this study, we first conducted single‐cell data analysis on 30 LUAD samples. The study design is illustrated in **Figure** **1**A. Principal component analysis (PCA) was used for dimensionality reduction, followed by t‐SNE for visualizing the clustering of these 30 samples (Figure 1B). Based on cell surface marker gene profiles provided by the Human Primary Cell Atlas, eight cell types were annotated, including two types of T cells, macrophages, epithelial cells, fibroblasts, B cells, mast cells, and endothelial cells (Figure 1C). The expression of cell surface markers and the percentage distribution of different cell types in each sample are shown in Figure S1A,B (Supporting Information). To identify key oncogenes in LUAD, we analyzed scRNA‐seq data to classify cells into tumor and normal groups, identifying differentially expressed genes between them (Figure S1C, Supporting Information). To further identify disease‐associated key genes from the differentially expressed genes, we utilized genetic data from the TRICL‐ILCCO consortium, comprising 29863 cases and 55586 controls, to perform GWAS. eQTL analysis was used to determine the relationship between gene expression and genetic variation, while pQTL analysis explored the relationship between genetic variation and protein expression levels. Among all differentially expressed genes, we identified 105 genes potentially associated with LUAD through eQTL analysis (Figure S1D, Supporting Information). Subsequently, through cis‐pQTL analysis with cumulative F‐statistics greater than 20, we identified NDRG1 as the gene most closely associated with LUAD progression (OR = 1.20; 95% CI = 1.06–1.37, *p* = 0.005). The corresponding MR analysis, based on clinical genetic data, further validated the causal relationship between NDRG1 and LUAD progression (Figure S1E, Supporting Information). Heterogeneity analysis using Cochran's Q test confirmed the stability and reliability of the results. All SNPs associated with NDRG1 were evenly distributed, indicating low heterogeneity and high homogeneity, which enhances the credibility of the results (Figure 1D). Pleiotropy analysis further confirmed the stability and reliability of the MR results for NDRG1 (Figure 1E). Therefore, we concluded that high expression of NDRG1 significantly increases the risk of developing LUAD.

After identifying NDRG1 as a key gene, we examined its behavior across various cancer types. Data from the TCGA Pan‐Cancer Atlas study, covering 22179 patients and 22802 samples across 36 cancer types, showed that NDRG1 frequently undergoes mutations, amplifications, and deep deletions in various cancers, including LUAD (Figure 1F). Furthermore, high expression of NDRG1 is closely associated with poor prognosis in various cancers (Figure S2A, Supporting Information), supporting its potential role in tumor progression. To better understand NDRG1's role in LUAD, we performed CNV analysis on epithelial cells using single‐cell data (Figure 1G). The results showed significant CNV changes in malignant cells compared to non‐malignant cells, indicating notable genomic instability. Additionally, we clustered epithelial cells into tumor and normal groups (Figure 1H), revealing that NDRG1 is mainly expressed in malignant cells (Figure 1I). The expression levels of NDRG1 in normal cells, NDRG1⁺ malignant cells, and NDRG1⁻ malignant cells showed that NDRG1⁺ malignant cells had significantly higher expression levels compared to both non‐malignant cells and NDRG1⁻ malignant cells, highlighting the distinct expression pattern in malignant cells (Figure 1J). Further analysis of clinical data from 1161 and 500 LUAD patients from two databases demonstrated that high NDRG1 expression significantly worsens the prognosis of LUAD patients (Figure 1K,L).

Our findings, consistent with previous studies,^[^ ^19^, ^20^ ^]^ highlight a significant association between NDRG1 expression and adverse prognosis in LUAD. To investigate the expression pattern of NDRG1, we analyzed tumor and adjacent non‐tumor tissues from 20 matched LUAD patients, collected at Zhongshan Hospital. qRT‐PCR revealed a marked upregulation of NDRG1 mRNA in tumor tissues compared to adjacent non‐tumor tissues (Figure 1M). Subsequently, Western blot analysis corroborated these findings, confirming a significant increase in NDRG1 protein levels in tumor samples (Figure 1N; Figure S1F, Supporting Information). Building upon these molecular findings, we examined the prognostic significance of NDRG1 by analyzing clinical data from 220 LUAD patients. Kaplan–Meier survival analysis demonstrated that high NDRG1 expression was associated with significantly poorer overall survival compared to patients with low expression (Figure 1O; log‐rank test, *p* = 0.0071). Moreover, multivariate Cox regression analysis identified tumor stage, lymph node metastasis, and NDRG1 expression as independent prognostic factors (Figure 1P). Specifically, lymph node metastasis (HR = 1.897, 95% CI: 1.113–3.234, *p* = 0.019), advanced tumor stage (III/IV) (HR = 2.203, 95% CI: 1.050–4.619, *p* = 0.037), and high NDRG1 expression (HR = 1.885, 95% CI: 1.209–2.937, *p* = 0.005) were all significantly associated with unfavorable clinical outcomes. These findings underscore the pivotal role of NDRG1 in LUAD progression and support its potential as an independent prognostic biomarker.

### 2.2 NDRG1 Promotes Glycolysis and Metabolic Reprogramming in LUAD

**Figure 2:**
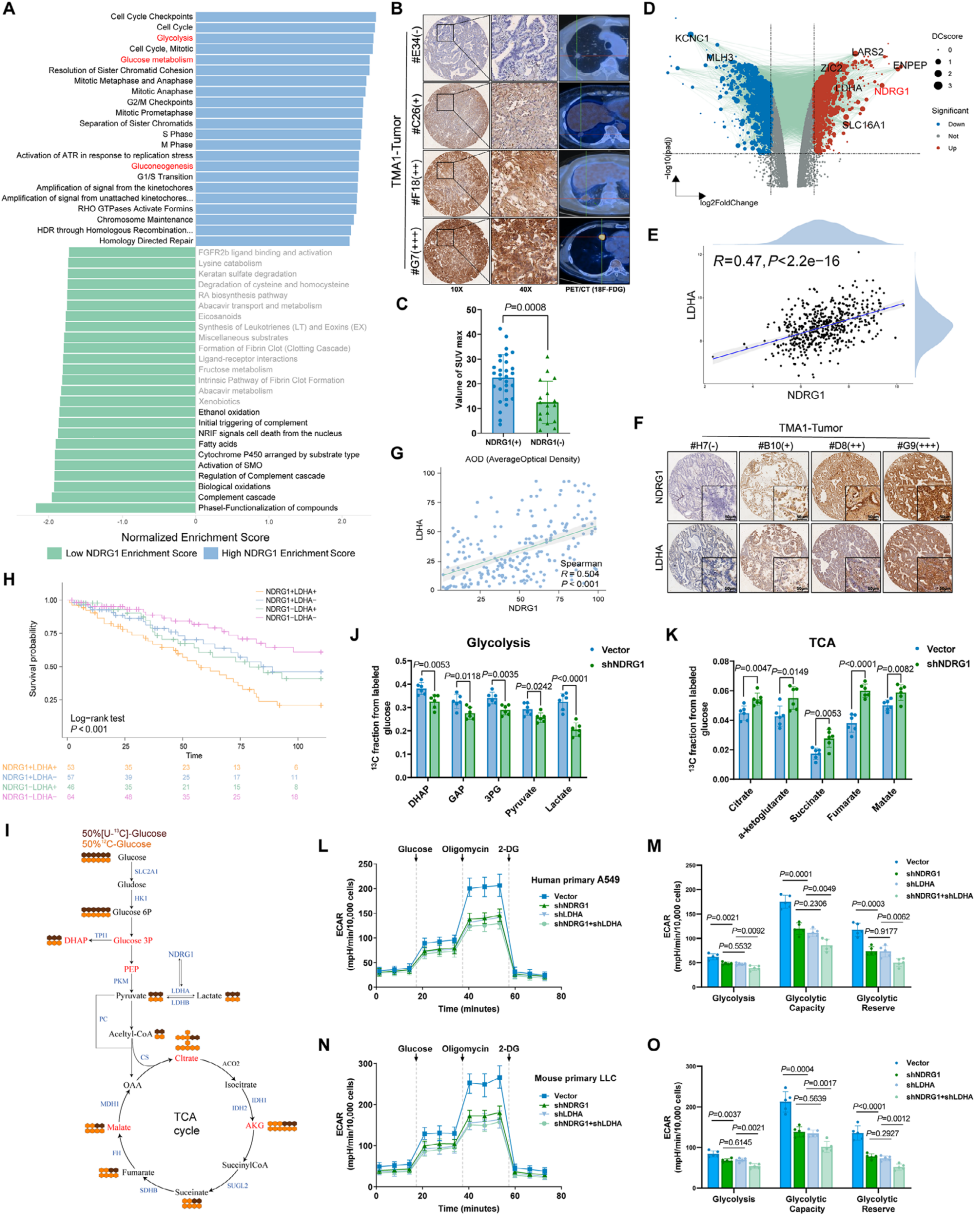
NDRG1 promotes glycolysis and metabolic reprogramming in LUAD. A) Reactome enrichment analysis results for high and low NDRG1 expression groups. B) Immunohistochemical staining of NDRG1 expression in LUAD patient tissue microarrays and 18F‐FDG PET/CT imaging results. C) Mann‐Whitney U test comparing the SUVmax distribution in NDRG1‐positive and NDRG1‐negative LUAD patients. D) Volcano plot showing the differential expression of NDRG1 and metabolism‐related genes. E) Scatter plot showing the correlation between NDRG1 and LDHA expression. F) Immunohistochemical staining analysis showing the expression levels of NDRG1 and LDHA in different samples: negative sample #H7, weak positive sample #B10, moderate positive sample #D8, and strong positive sample #G9. G) Average optical density (AOD) analysis of the correlation between NDRG1 and LDHA expression. H) Survival analysis comparing the survival rates of patients with combined high expression of NDRG1 and LDHA. I) Schematic molecular roadmap briefly outlining the landscape of glucose metabolism in mammalian cells. J,K) GC‐MS analysis results related to glycolysis and the TCA cycle, showing the effect of NDRG1 knockdown on the concentration of glycolysis and TCA cycle intermediates (n=6). L,M) ECAR response curve and quantitative analysis (Glycolysis, Glycolytic Capacity, Glycolytic Reserve) of A549 cells under different conditions (Vector, shNDRG1, shLDHA, shNDRG1+shLDHA) after treatment with glucose, oligomycin, and 2‐DG (n=5). N,O) ECAR response curve and quantitative analysis (Glycolysis, Glycolytic Capacity, Glycolytic Reserve) of LLC cells under different conditions (Vector, shNDRG1, shLDHA, shNDRG1+shLDHA) after treatment with glucose, oligomycin, and 2‐DG (n=5). Differences were considered statistically significant at *p* < 0.05.

To systematically elucidate the mechanisms underlying the poor prognosis associated with elevated NDRG1 expression in LUAD, we conducted a functional enrichment analysis of NDRG1 expression using TCGA data. This analysis identified significant associations between elevated NDRG1 expression and pathways related to cell cycle regulation, cell division, glycolysis, glucose metabolism, and gluconeogenesis (**Figure** **2**A), suggesting that NDRG1 plays a pivotal role in metabolic reprogramming. Further examination using 18F‐FDG PET/CT imaging demonstrated a positive correlation between maximum standardized uptake value (SUVmax) and NDRG1 expression levels (Figure 2B,C), linking high NDRG1 expression with increased glucose metabolic activity in tumors. Volcano plot analysis of differentially expressed genes showed that NDRG1 is markedly upregulated in tumor tissues, alongside several key metabolic genes, including LDHA, SLC16A1, PKM, HK1, and LDHB (Figure 2D; Figure S2B, Supporting Information). NDRG1 exhibited a significant positive correlation with these metabolic genes, particularly LDHA (R = 0.47, *p* < 0.001) (Figure 2E; Figure S2C, Supporting Information). This relationship was further validated by immunohistochemical (IHC) staining and Average Optical Density (AOD) analysis, both of which confirmed a strong correlation between NDRG1 and LDHA expression (R = 0.504, *p* < 0.001) (Figure 2F,G). Survival analysis emphasized the clinical relevance of these findings, demonstrating that high co‐expression of NDRG1 and LDHA correlates with significantly poorer survival outcomes in LUAD patients (Figure 2H).

To better understand the metabolic pathways influenced by NDRG1, we constructed a pathway diagram outlining the progression from glucose transport to pyruvate production and the initial stages of the Tricarboxylic Acid (TCA) cycle, focusing on critical intermediates such as G6P, DHAP, and G3P, as well as key regulatory proteins (Figure 2I).^[^ ^14^ ^]^ Quantitative expression analysis across multiple LUAD cell lines, including A549, LLC, PC9, H1299, and H1975, revealed significant variability in NDRG1 expression. A549 cells exhibited the highest levels, followed by LLC (Figure S3A, Supporting Information). Western blot analysis further confirmed these findings, showing significantly higher protein expression in A549 and LLC cells (Figure S3B, Supporting Information). Therefore, we performed experimental analyses in these two cell lines. In A549 cells subjected to NDRG1 knockdown, GC‐MS analysis indicated alterations in crucial metabolic intermediates in both glycolysis and the TCA cycle, including citrate, isocitrate, α‐ketoglutarate, pyruvate, and lactate (Figure 2J,K). These changes suggest that NDRG1 promotes a metabolic shift toward glycolysis by modulating key glycolytic intermediates. To investigate the functional role of NDRG1 in glycolysis, we used RNA interference to suppress its expression. Results showed no significant differences in the mRNA expression of key glycolytic enzymes following NDRG1 knockdown (Figure S3C, Supporting Information). Extracellular acidification rate (ECAR) analysis revealed that glycolytic capacity was significantly reduced in A549 and LLC cells after NDRG1 knockdown, underscoring NDRG1's role in maintaining high glycolytic flux (Figure 2L–O). Additionally, oxygen consumption rate (OCR) analysis showed that NDRG1 knockdown led to a significant increase in oxidative phosphorylation capacity, indicating that NDRG1 normally suppresses mitochondrial function to direct cells toward glycolysis (Figure S3D–G, Supporting Information). When both NDRG1 and LDHA were simultaneously knocked down, a more pronounced reduction in ECAR was observed compared to single knockdowns, demonstrating a synergistic effect between NDRG1 and LDHA in promoting glycolysis. Conversely, OCR showed a substantial increase with dual knockdown, particularly in maximal respiratory capacity, suggesting that NDRG1 and LDHA together play a key role in repressing oxidative phosphorylation.

### 2.3 NDRG1 and LDHA Functionally Cooperate to Promote Glycolysis

**Figure 3:**
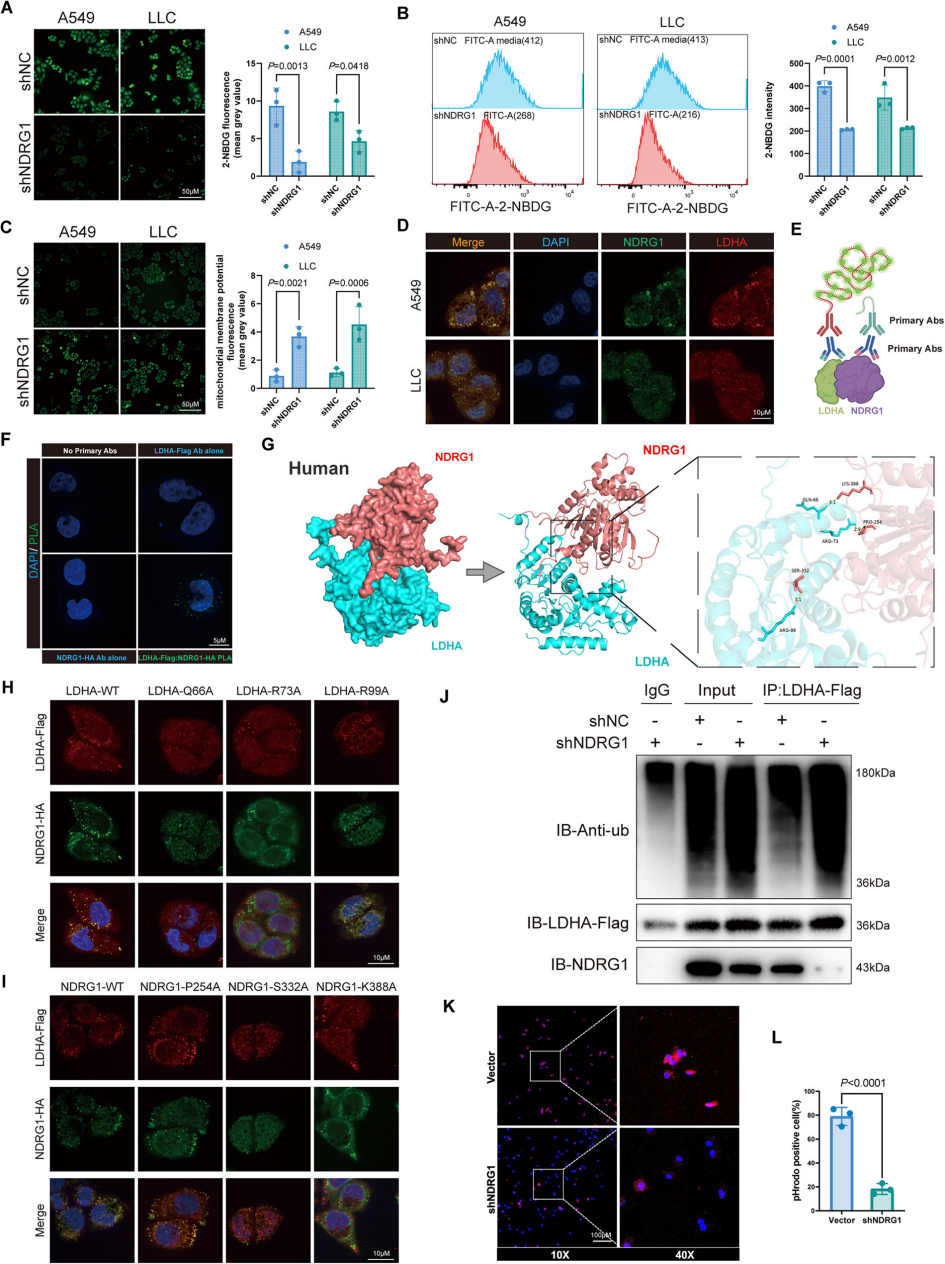
NDRG1 and LDHA functionally cooperate to promote glycolysis. A,B) Fluorescence microscopy images and flow cytometry analysis of 2‐NBDG glucose uptake, showing glucose uptake results in A549 and LLC cells with normal and knocked‐down NDRG1 expression. C) Rhodamine 123 fluorescence images showing mitochondrial membrane potential results in A549 and LLC cells with normal and knocked‐down NDRG1 expression. D) Immunofluorescence colocalization images showing NDRG1 and LDHA in A549 and LLC cells, highlighting their spatial overlap. E,F) PLA images indicating direct interactions between NDRG1 and LDHA, as evidenced by green fluorescent signals. G) Molecular docking analysis showing the complex between human NDRG1 and LDHA, with a focus on interaction sites. H) Immunofluorescence colocalization studies illustrating the effects of LDHA mutations on its interaction with NDRG1. I) Immunofluorescence colocalization showing the impact of NDRG1 mutations on its colocalization with LDHA. J) Immunoprecipitation (IP) results displaying the ubiquitination levels of LDHA in NDRG1 knockdown versus control cells (n=3). K) Quantitative immunofluorescence analysis showing fluorescence quantification results under different NDRG1 expression conditions (n=3). L) pHrodo staining experiment showing the percentage of cells under different NDRG1 expression conditions (n=3). Differences were considered statistically significant at *p* < 0.05.

To investigate the cooperative role of NDRG1 and LDHA in glycolysis, we first examined how NDRG1 affects glucose uptake and mitochondrial function in tumor cells. Fluorescence microscopy and flow cytometry analysis of 2‐NBDG glucose uptake showed that knockdown of NDRG1 in A549 and LLC cells led to a significant reduction in glucose uptake (**Figure** **3**A,B), suggesting that NDRG1 enhances glucose entry, a critical step for maintaining high glycolytic flux. Additionally, Rhodamine 123 fluorescence imaging revealed an increase in mitochondrial membrane potential in NDRG1 knockdown cells (Figure 3C), indicating that when NDRG1 is present, it suppresses mitochondrial function, likely promoting a shift toward glycolysis for energy production. Protein‐protein interaction (PPI) network analysis revealed that NDRG1, LDHA, and other glycolysis‐related genes, such as PKM, HIF1A, and ENO1, form a complex network, suggesting a coordinated role in promoting glycolytic metabolism (Figure S3H, Supporting Information). Based on these findings, we explored whether NDRG1 collaborates with LDHA, a key enzyme in glycolysis, to regulate this metabolic shift. Immunofluorescence co‐localization experiments demonstrated a substantial spatial overlap between NDRG1 and LDHA in A549 and LLC cells (Figure 3D), suggesting that they might work together to facilitate glycolysis. Additional evidence from the proximity ligation assay (PLA) confirmed a direct intracellular interaction between NDRG1 and LDHA, as indicated by prominent green fluorescence signals that were absent in the control group (Figure 3E,F). To further validate this interaction, molecular docking analysis identified specific binding sites, Lys388 in NDRG1 and Arg73 in LDHA, which were also observed in murine models (Figure 3G; Figure S3I, Supporting Information). Mutation of these residues reduced co‐localization in immunofluorescence experiments, highlighting their critical role in facilitating the formation of the NDRG1‐LDHA complex (Figure 3H,I).

Next, we examined whether NDRG1 affects LDHA stability and functionality within the glycolytic pathway. Preliminary analysis suggested that ubiquitination of LDHA, particularly at residue K76, is critical for its stability and enzymatic activity (Figure S3J, Supporting Information). Immunoprecipitation (IP) assays showed that LDHA ubiquitination levels were significantly elevated in the NDRG1 knockdown group, especially in cells expressing LDHA‐Flag (Figure 3J). This finding suggests that NDRG1 reduces LDHA's susceptibility to ubiquitination, thereby stabilizing LDHA to enhance its role in glycolysis. Further immunofluorescence staining indicated that NDRG1 knockdown led to a reduction in LDHA expression in A549 cells (Figure 3K). Additionally, pHrodo staining showed that the control group exhibited a more acidic intracellular environment compared to the NDRG1 knockdown group (Figure 3L). This difference in acidity suggests a reduction in lactate production following NDRG1 knockdown, hinting that NDRG1's role in stabilizing LDHA may drive lactate production as a downstream effect of enhanced glycolysis.

### 2.4 NDRG1 Promotes Lactate Accumulation, M2 Polarization, and Inhibits CD8^+^ T Cell Function

**Figure 4:**
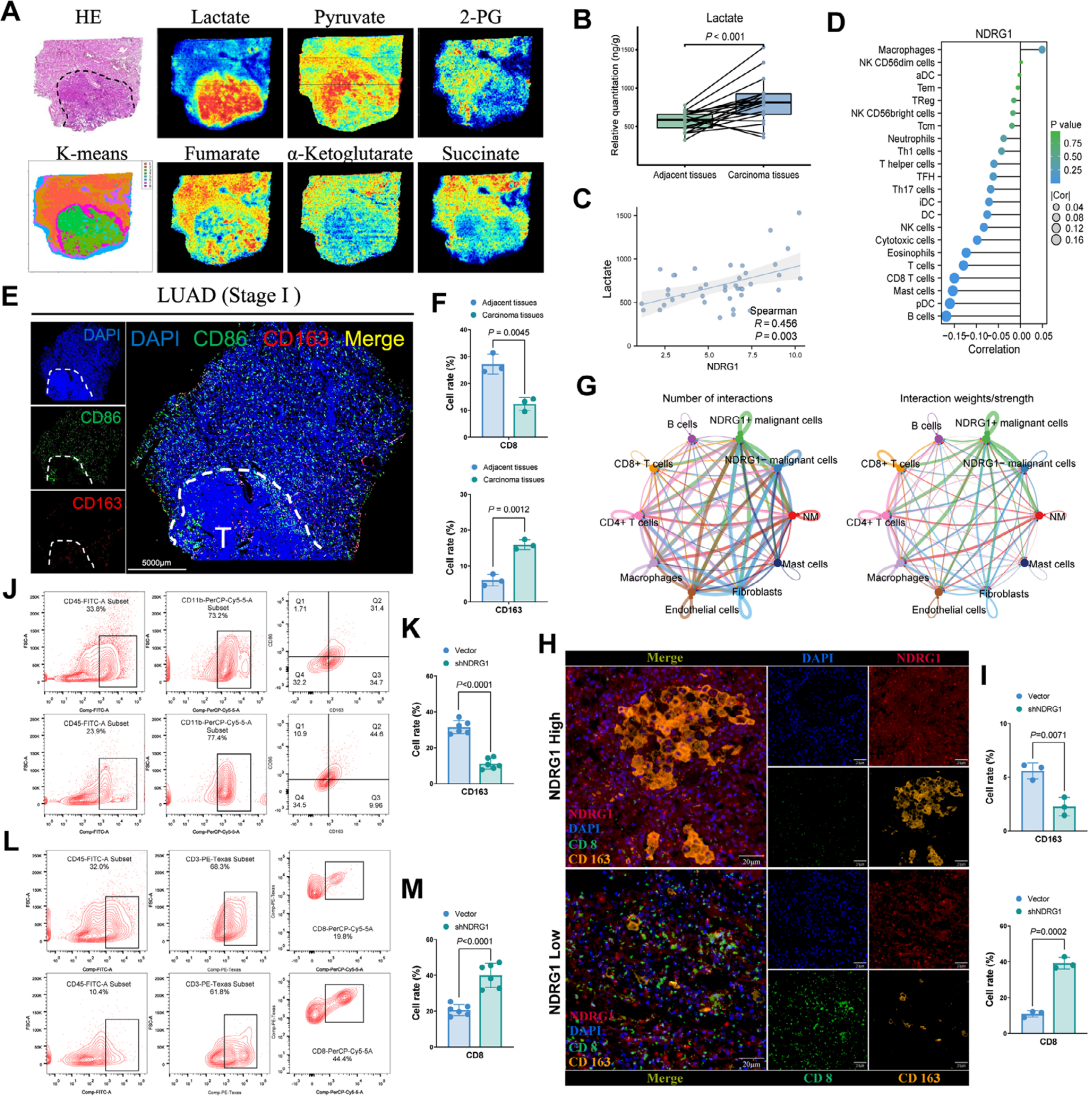
NDRG1 promotes lactate accumulation, M2 polarization, and inhibits CD8+ T cell function. A) Spatial metabolomics analysis showing the distribution of lactate, pyruvate, 2‐phosphoglycerate (2‐PG), fumarate, α‐ketoglutarate, and succinate in resected LUAD slices and adjacent normal tissues. B) Quantification of lactate levels in tumor tissues and adjacent normal tissues (n=10). C) Correlation analysis between lactate concentration and NDRG1 expression. D) Correlation analysis of NDRG1 expression with different immune cell subsets in the TCGA database. E) Immunofluorescence imaging showing the expression of CD163 (M2 macrophage marker) and CD8 (T cell marker) in resected LUAD slices and adjacent normal tissues. F) Quantification of CD8⁺ T cell proportion and CD163 expression from immunofluorescence images (n=3). G) Cell–cell communication analysis illustrating interactions between NDRG1⁺/⁻ cancer cells and various immune cells in lung adenocarcinoma samples. H) Immunofluorescence imaging of NDRG1 and immune markers in the tumor microenvironment. I) Quantification of NDRG1 expression and immune markers in the tumor microenvironment (n=3). J,K) Flow cytometry analysis and quantification of CD45⁺CD163⁺ M2 macrophage distribution under normal and NDRG1 knockdown conditions (n=6). L,M) Flow cytometry analysis and quantification of CD45⁺CD8⁺ T cell distribution under normal and NDRG1 knockdown conditions (n=6). Differences were considered statistically significant at *p* < 0.05.

In our prior experiments, we found that NDRG1 influences tumor cell metabolic functions by regulating glycolysis and xoxidative metabolism, particularly in A549 cells, where it synergistically interacts with LDHA to promote lactate production and alter the acidic microenvironment of tumor cells. To further explore how NDRG1‐mediated metabolic changes impact the immune microenvironment, we conducted a more in‐depth investigation. First, using hematoxylin and eosin (H&E) staining on resected LUAD samples, we were able to distinguish between tumor and normal tissues. Spatial metabolomics analysis revealed significantly higher levels of key metabolic intermediates, such as lactate and pyruvate, in the tumor regions compared to adjacent normal tissues, confirming enhanced glycolysis (**Figure** **4**A,B). Notably, lactate levels showed a positive correlation with NDRG1 expression (R = 0.456, *p* = 0.003), suggesting that NDRG1 plays a critical role in driving glycolysis and lactate production in tumors (Figure 4C). Analysis of immune data from the TCGA database indicated that elevated NDRG1 expression was associated with an increased presence of macrophages and a reduction in immune effector cells, including CD8^+^ T cells, suggesting that NDRG1 may contribute to the formation of an immunosuppressive TME (Figure 4D).

aImmunofluorescence analysis further confirmed that regions with higher NDRG1 expression exhibited a significant increase in CD163‐positive M2 macrophages and a decrease in CD8^+^ T cells (Figure 4E,F). To directly investigate intercellular interactions within the TME, we performed single‐cell communication analysis. The results showed that NDRG1‐positive malignant epithelial cells had stronger interactions with macrophages and weaker interactions with CD8^+^ T cells, reinforcing the idea that NDRG1 promotes immunosuppressive cell interactions while suppressing cytotoxic immune responses (Figure 4G). In murine models, these findings were consistent with the human data. Immunofluorescence revealed that tumors with elevated NDRG1 expression had an increased number of CD163^+^ macrophages and a reduced number of CD8^+^ T cells (Figure 4H,I). Flow cytometry analysis further supported these results, demonstrating an increase in M2 macrophages and a decrease in CD8^+^ T cells in tumors with high NDRG1 expression (Figure 4J–M). Collectively, these findings highlight the critical role of NDRG1 in orchestrating an immunosuppressive TME by driving lactate accumulation, promoting M2 macrophage polarization, and inhibiting CD8^+^ T cell function, thereby facilitating tumor progression.

### 2.5 NDRG1‐Induced Lactate Production Drives Immune Suppression Function

**Figure 5:**
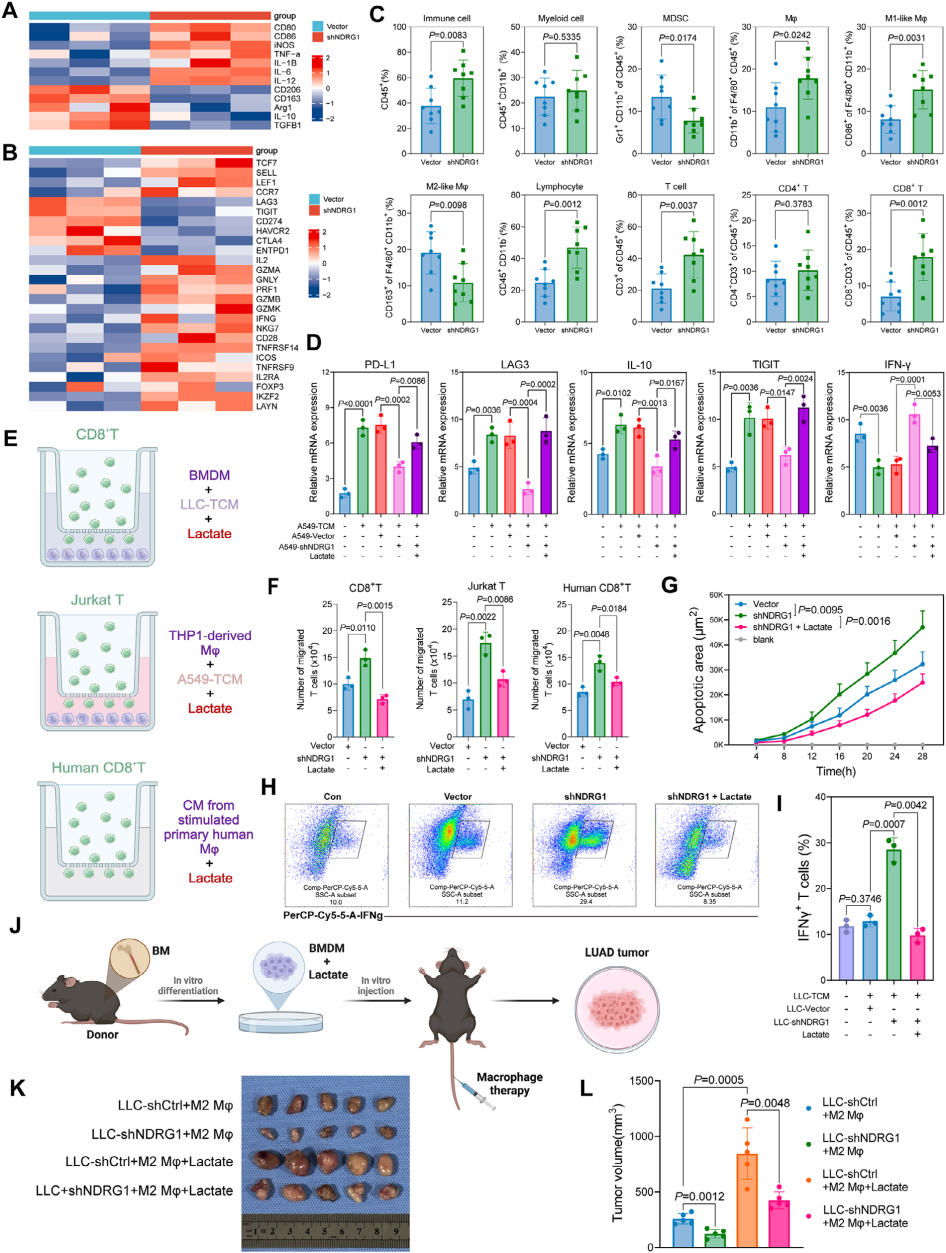
NDRG1‐induced lactate production drives immune suppression. A)Heatmap analysis of macrophage marker mRNA expression in NDRG1 knockdown (shNDRG1) and control (vector) A549 cells co‐cultured with THP‐1 macrophages for 12 h in tumor‐conditioned medium (TCM). B) Heatmap analysis of CD8⁺ T cell marker mRNA expression in NDRG1 knockdown (shNDRG1) and control (vector) A549 cells co‐cultured with macrophages and CD8⁺ T cells for 12 h. C) Flow cytometry‐based analysis of immune cell populations in A549 tumors from NDRG1⁺ and NDRG1⁻ mice. Tumors were harvested 14 days post‐inoculation. The proportions of total immune cells, myeloid cells, myeloid‐derived suppressor cells, macrophages, M1‐like macrophages, M2‐like macrophages, lymphocytes, total T cells, CD4⁺ T cells, and CD8⁺ T cells were quantified (n=8). D) Relative mRNA expression levels of PD‐L1, LAG3, IL10, TIGIT, and IFNG in untreated A549 cells (A549‐TCM), control A549 cells (A549‐vector), NDRG1 knockdown A549 cells (A549‐shNDRG1), and lactate‐treated A549 cells (n=3). E) Effects of NDRG1 knockdown and lactate treatment on the migration of CD8⁺ T cells and Jurkat T cells（Created in BioRender）. F) Flow cytometry‐based characterization of immune cell populations in LLC tumors from NDRG1⁺ and NDRG1⁻ mice, harvested 14 days post‐inoculation, including myeloid cells, MDSCs, macrophages, M1‐like macrophages, M2‐like macrophages, CD8⁺ T cells, and CD4⁺ T cells (n=3). G–I) Effects of NDRG1 knockdown on apoptotic area (G) and IFNγ⁺ T cells proportion (H, I) under various treatments (n = 3). J) Schematic representation of the subcutaneous tumor model（Created in BioRender）. K) Representative tumor images from mice under different treatment conditions: LLC‐shCtrl + M2 macrophages, LLC‐shNDRG1 + M2 macrophages, LLC‐shCtrl + M2 macrophages + lactate, and LLC‐shNDRG1 + M2 macrophages + lactate. L) Quantification of tumor volume (mm^3^) in the indicated groups (n=5). Differences were considered statistically significant at *p* < 0.05.

To explore the role of NDRG1‐induced lactate production in shaping the immunosuppressive TME, we conducted a series of experiments. First, we found that NDRG1 knockdown in A549 cells significantly reduced lactate levels in the tumor–conditioned media (TCM). When THP‐1‐derived macrophages were cultured with TCM from shNDRG1 cells, RNA sequencing analysis revealed a macrophage polarization shift toward a pro‐inflammatory M1 phenotype, characterized by upregulated expression of M1‐associated markers (CD80, CD86, iNOS, TNF‐α, IL‐1β, IL6, IL12) and downregulated expression of M2‐associated markers (CD206, CD163, ARG1, IL10, TGFB1) (**Figure** **5**A). Next, these macrophages were co‐cultured with CD8⁺ T cells for 12 h to assess their immunomodulatory effects. CD8⁺ T cells in the shNDRG1 TCM group exhibited enhanced activation, evidenced by increased expression of activation markers (IL‐2, CD28, ICOS) and cytotoxic effectors (GZMA, GZMB, PRF1, IFN‐γ, NKG7), along with downregulation of immune checkpoint genes (CTLA4, TIGIT, LAG3, CD274, HAVCR2) (Figure 5B). These transcriptional changes were further validated by PCR analysis, confirming the modulation of both macrophage polarization and T cell activation (Figure S4A,B, Supporting Information). Flow cytometry analysis of immune cells extracted from C57BL/6 mice tumors 14 days post‐injection also supported these findings, showing a reduction in myeloid‐derived suppressor cells (MDSCs) and M2 macrophages in the shNDRG1 group, coupled with an increase in M1‐like macrophages and CD8^+^ T cells (Figure 5C).

Further investigation revealed that NDRG1 promotes immunosuppression by driving lactate accumulation, which regulates the expression of genes critical to immune function. Gene expression analysis demonstrated that in shNDRG1‐treated cells, immunosuppressive genes (PD‐L1, LAG3, IL‐10, TIGIT) were downregulated, while the immune‐activating cytokine IFNγ was upregulated. However, when exogenous lactate was added, these immune‐suppressive genes were re‐expressed, and IFNγ was downregulated, restoring the immunosuppressive environment (Figure 5D). Transwell migration and T‐cell activation assays confirmed these findings. Knockdown of NDRG1 enhanced the migration and activation of both CD8⁺ T cells and Jurkat T cells, whereas lactate treatment reversed these effects (Figure 5E,F). Tumor cell apoptosis was significantly increased in the shNDRG1 group, but this enhancement was partially mitigated by lactate (Figure 5G). Similarly, shNDRG1 elevated the proportion of IFNγ⁺ T cells, an effect that was again partially suppressed by lactate administration (Figure 5H,I).

To investigate the role of NDRG1‐induced lactate production in tumor progression, we established a subcutaneous LLC tumor model (Figure 5J). LLC cells transduced with either shCtrl or shNDRG1 were subcutaneously injected into the flanks of mice. Concurrently, M2 macrophages pretreated with or without lactate were administered via tail vein injection. As shown in Figure 5K,L, tumors in the LLC‐shNDRG1+M2 macrophage group were significantly smaller than those in the LLC‐shCtrl+M2 macrophage group, indicating that NDRG1 knockdown effectively suppresses tumor growth. Furthermore, in both the LLC‐shCtrl and LLC‐shNDRG1 groups, lactate‐treated M2 macrophages promoted further tumor progression compared to untreated M2 macrophages. These findings suggest that lactate enhances the tumor–promoting activity of M2 macrophages. Given these results, we propose that lactate may induce lactylation modifications in M2 macrophages, thereby enhancing their immunosuppressive activity and further driving tumor progression. This hypothesis forms the basis for our subsequent experiments, to explore the role of lactylation modifications in mediating the effects of lactate on M2 macrophages.

### 2.6 Lactate Regulates Macrophage H3K18 Lactylation

**Figure 6:**
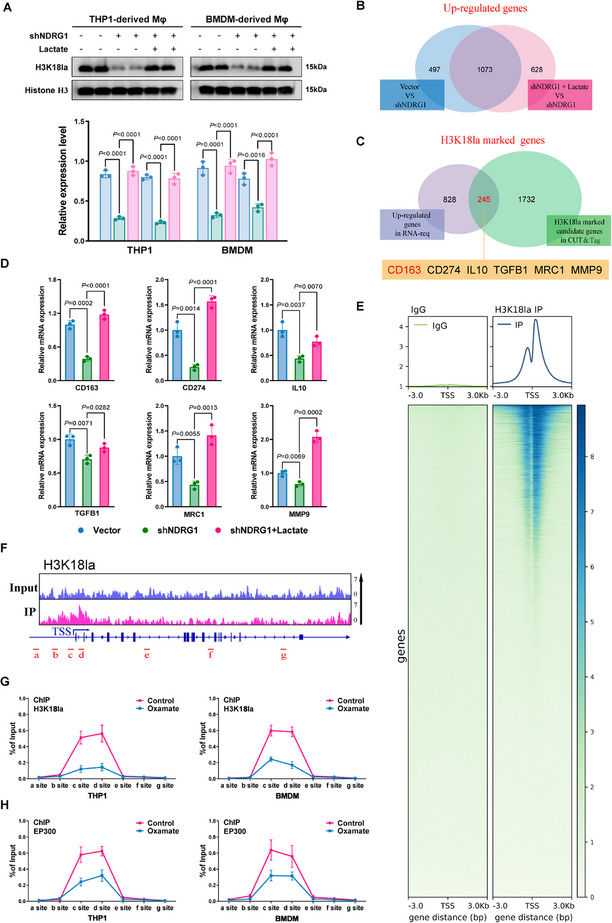
NDRG1 regulates macrophage H3K18 lactylation. A) Western blot analysis of H3K18la and Histone H3 levels in THP1‐derived and BMDM‐derived macrophages under control (Vector), NDRG1 knockdown (shNDRG1), and shNDRG1 + Lactate conditions (n=3). B) Venn diagram illustrating the overlap of upregulated genes between shNDRG1 and shNDRG1 + Lactate conditions. C) Venn diagram showing the intersection of H3K18la‐marked genes identified through RNA‐seq and CUT&Tag analyses. D) RT‐qPCR analysis of mRNA expression levels for CD163, PDCD1, MMP9, TGFB1, MRC1, and IL10 in THP1‐derived and BMDM‐derived macrophages under Vector, shNDRG1, and shNDRG1 + Lactate conditions (n=3). E) ChIP‐seq analysis of H3K18la enrichment in macrophage promoter regions, including transcription start sites (TSS). F) ChIP‐seq profile showing H3K18la signals at the CD163 gene locus. G) ChIP‐qPCR analysis of H3K18la status at the CD163 genomic region in THP1 and BMDM cells (n=3). H) ChIP‐qPCR analysis of EP300 binding at the CD163 promoter region in THP1 and BMDM cells (n=3). Differences were considered statistically significant at *p* < 0.05.

Based on our previous findings, we investigated whether tumor cell‐derived NDRG1 and lactate influence histone H3K18 lactylation (H3K18la) in macrophages and its role in regulating immunosuppressive genes. First, we knocked down NDRG1 in tumor cells, collected their TCM, and co‐cultured it with THP‐1‐derived macrophages or bone marrow‐derived macrophages (BMDMs). Western blot results showed that NDRG1 knockdown in tumor cells significantly reduced H3K18la levels in macrophages, whereas treatment with exogenous lactate partially restored H3K18la enrichment (**Figure** **6**A). This indicates that tumor cell‐derived NDRG1 regulates H3K18la levels in macrophages via lactate, thereby affecting macrophage function through epigenetic mechanisms. Next, we investigated the effects of tumor cell‐derived NDRG1 and lactate on gene expression in macrophages through RNA‐seq. RNA‐seq analysis revealed 1073 upregulated genes in macrophages co‐cultured with NDRG1 knockdown tumor cells compared to the lactate‐treated group (Figure 6B). To explore whether these upregulated genes are regulated by epigenetic modifications, we integrated CUT&Tag data and analyzed H3K18la enrichment at promoter regions in macrophages. Intersection analysis identified 245 genes with H3K18la‐enriched promoters, including immunosuppressive genes such as CD163, PD‐L1, IL10, TGFB1, MRC1, and MMP9 (Figure 6C). The regulation of CD163 is crucial for M2 macrophage polarization, while the other immunosuppressive genes contribute to the establishment and reinforcement of an immunosuppressive tumor microenvironment. qPCR analysis showed that macrophages co‐cultured with NDRG1 knockdown tumor cells exhibited significantly decreased expression of these immunosuppressive genes, while lactate treatment partially restored their expression (Figure 6D). These results further support the notion that tumor‐derived NDRG1, through lactate‐mediated histone modifications, regulates a network of genes in macrophages to promote immunosuppression within the TME.

To further explore the role of H3K18la in regulating CD163 and other immunosuppressive genes, we performed a ChIP‐seq analysis. The results showed significant H3K18la enrichment at promoter regions, particularly near the transcription start sites (TSS), of macrophage genes (Figure 6E). Further analysis revealed significant H3K18la signals at the promoters of CD163 and other immunosuppressive genes (Figure 6F; Figure S4C, Supporting Information), suggesting that tumor‐derived NDRG1 regulates the expression of these genes through lactate‐dependent H3K18la modifications. To verify the impact of glycolysis on H3K18la enrichment through lactate accumulation, we treated macrophages with a glycolysis inhibitor and performed ChIP‐qPCR to assess H3K18la levels at the CD163 promoter region. The results showed that the glycolysis inhibitor significantly reduced H3K18la levels at the CD163 promoter (Figure 6G,H) and also decreased the binding of the histone lactylation writer EP300 at this region. This suggests that glycolysis, through lactate and H3K18la modifications, regulates the expression of CD163 and other immunosuppressive genes, further promoting M2 macrophage polarization and immunosuppressive function. In summary, NDRG1 promotes lactate accumulation and histone H3K18 lactylation, which specifically regulate CD163 expression and facilitate M2 macrophage polarization. Additionally, through lactate‐dependent histone lactylation modifications, NDRG1 regulates other immunosuppressive genes such as PD‐L1, MMP9, TGFB1, IL10, and MRC1. The coordinated regulation of these genes further enhances the immunosuppressive effects of macrophages within the TME, highlighting the critical role of NDRG1 and lactate in tumor immune evasion.

### 2.7 Targeting NDRG1 in Combination with αPD‐L1 Therapy Can Improve Treatment Outcomes in LUAD

**Figure 7:**
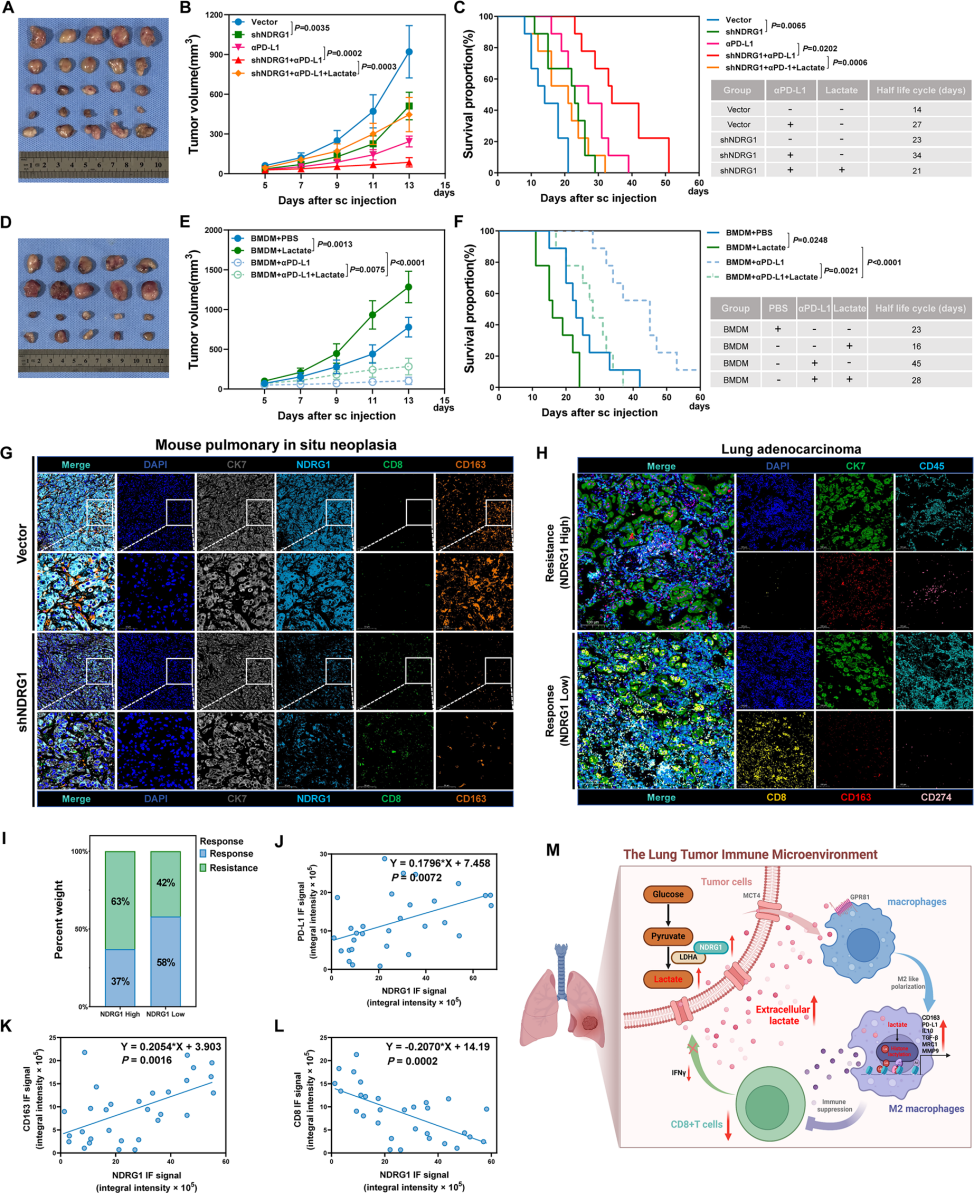
Targeting NDRG1 in combination with αPD‐L1 therapy can improve treatment outcomes in LUAD. A–C) The combined effect of NDRG1 knockdown and αPD‐L1 treatment on tumor volume and survival rate. Experimental groups include Vector control, shNDRG1 group, αPD‐L1 group, shNDRG1+αPD‐L1 group, and shNDRG1+Lactate+αPD‐L1 group (n=5). D–F) The combined effect of Lactate and αPD‐L1 treatment on tumor volume and survival rate. Experimental groups include BMDM+PBS control group, BMDM+Lactate group, BMDM+αPD‐L1 group, and BMDM+Lactate+αPD‐L1 group (n=5). G) Immunofluorescence staining of lung in situ tumors in mice from Vector control and shNDRG1 groups, marking DAPI, CK7, NDRG1, CD8, and CD163. H) Immunofluorescence staining of lung adenocarcinoma tissues in patients with low (responsive) and high (resistant) NDRG1 expression. Markers include DAPI, CK7, CD45, CD8, CD163, and PD‐L1. I) Percentage of immunotherapy response related to NDRG1 expression. J–L) Correlation between NDRG1 expression and immune cell markers (CD8, CD163, and PD‐L1) expression. M) A schematic model depicting the tumor microenvironment, showing NDRG1‐mediated lactate increase leading to an immunosuppressive microenvironment（Created in BioRender）. Differences were considered statistically significant at *p* < 0.05.

Given our findings that NDRG1 promotes an immunosuppressive tumor microenvironment through lactate‐mediated immune cell regulation, we next explored the therapeutic potential of combining NDRG1 targeting with αPD‐L1 therapy in a LUAD model. Our results show that both NDRG1 knockdown and αPD‐L1 monotherapy (200 µg per mouse, administered intraperitoneally every three days for a total of five doses) significantly inhibited tumor growth. Notably, their combination produced a stronger anti‐tumor effect, suggesting a synergistic therapeutic response (**Figure** **7**A,B). However, lactate supplementation (1.68 g kg^−1^ day^−1^ sodium L‐lactate, administered via subcutaneous injection throughout the treatment period) reversed the therapeutic benefit of the combination treatment, leading to increased tumor volumes. This highlights the essential role of lactate in maintaining an immunosuppressive tumor microenvironment. Survival analysis further supported the superiority of the combination strategy: while NDRG1 knockdown or αPD‐L1 alone extended survival, their combination provided the most pronounced benefit, which was notably diminished by lactate treatment (Figure 7C). Similarly, in BMDM‐based experiments, αPD‐L1 therapy combined with BMDM treatment significantly suppressed tumor growth, but this effect was weakened when lactate was introduced (Figure 7D,E). Survival analysis mirrored this trend, with the BMDM + αPD‐L1 group showing the longest survival, which was shortened by lactate supplementation (Figure 7F). These findings highlight the clinical potential of targeting NDRG1 to enhance immunotherapy efficacy, demonstrating a superior anti‐tumor effect when combined with αPD‐L1 therapy. Given the critical role of NDRG1 in shaping the TME, we next examined how its expression influences immune cell composition and function in LUAD.

Immunofluorescence staining provided further insights into the immune landscape of LUAD. In a mouse model of pulmonary in situ neoplasia, high NDRG1 expression was associated with reduced CD8^+^ T cell infiltration and increased numbers of CD163^+^ M2 macrophages, indicative of an immunosuppressive microenvironment. NDRG1 knockdown reversed this trend, leading to increased infiltration of CD8^+^ T cells and reduced numbers of CD163^+^ macrophages, suggesting that NDRG1 plays a crucial role in maintaining immune suppression during early‐stage lung tumor development (Figure 7G). These findings highlight the potential of targeting NDRG1 to enhance anti‐tumor immune responses by modulating the balance between immune effector and suppressor cells. Clinical sample analysis from lung adenocarcinoma patients further emphasized the importance of NDRG1 as a therapeutic target. Patients with low NDRG1 expression showed increased CD8^+^ T cell infiltration and responded better to immunotherapy, whereas patients with high NDRG1 expression exhibited reduced CD8^+^ T cell infiltration, increased numbers of CD163^+^ macrophages, and elevated PD‐L1 expression, indicating a stronger immunosuppressive tumor microenvironment (Figure 7H). Strikingly, 58% of patients with low NDRG1 expression responded to immunotherapy, compared to only 37% of those with high NDRG1 expression, suggesting that high NDRG1 expression is associated with immunotherapy resistance (Figure 7I). Correlation analysis confirmed that NDRG1 expression was positively correlated with PD‐L1 and CD163 levels and negatively correlated with CD8^+^ T cell infiltration (Figure 7J–L), further emphasizing the role of NDRG1 in immune regulation and lactate metabolism. A schematic model (Figure 7M) illustrates how NDRG1‐mediated lactate accumulation promotes M2 macrophage polarization and inhibits CD8^+^ T cell activity, further enhancing the immunosuppressive tumor microenvironment and reducing the efficacy of immunotherapy. In conclusion, these findings emphasize the critical role of NDRG1 in shaping the tumor microenvironment through lactate‐mediated mechanisms, and targeting NDRG1 in combination with strategies to modulate lactate metabolism may significantly improve the effectiveness of immunotherapy in treating lung adenocarcinoma.

## 3 Discussion

The accumulation of lactate within TME is considered a key driver of immunosuppression and tumor progression.^[^ ^7^, ^8^, ^9^ ^]^ Tumor cells enhance glycolysis, leading to the production of large amounts of lactate, which not only supports tumor growth and survival but also impairs antitumor immune responses by altering immune cell function.^[^ ^10^ ^]^ Lactate facilitates the polarization of macrophages toward the M2 phenotype, which is typically associated with anti‐inflammatory responses, tissue repair, and tumor promotion.^[^ ^12^, ^13^ ^]^ Simultaneously, lactate diminishes the activity of CD8^+^ T cells, further weakening the immune system's surveillance against tumors.^[^ ^14^, ^15^ ^]^ These findings suggest that targeting lactate metabolism could be an effective strategy to overcome immunosuppression. By modulating lactate production and its effects, it may be possible to reverse the acidic TME, restore immune cell function, and enhance antitumor immunity.^[^ ^16^, ^21^ ^]^ Therefore, the development of immunotherapeutic strategies targeting lactate metabolism has emerged as a promising direction in cancer immunotherapy.

In this study, we explored the key genes driving LUAD progression from two critical dimensions: molecular and clinical. On the molecular level, we utilized scRNA‐seq to analyze TME and identify differentially expressed genes that are pivotal in tumor biology. On the clinical level, we integrated these molecular findings with large‐scale genetic data from LUAD patients, using MR to uncover causal relationships between these genes and LUAD progression. Through this comprehensive approach, we identified NDRG1 as a key regulator involved in metabolic reprogramming and immune regulation within the TME. Our findings demonstrate that NDRG1 contributes to enhanced glycolytic activity in tumor cells, leading to increased lactate production. This metabolic shift results in the accumulation of lactate in the TME, which exerts widespread effects on both tumor and immune cells. Lactate not only drives the metabolic reprogramming that polarizes macrophages toward an immunosuppressive M2 phenotype but also further reinforces this polarization through histone lactylation. Specifically, lactate accumulation in the TME drives M2 macrophage polarization and, through lactylation, modulates the expression of immune regulatory genes. This modification enhances the expression of genes associated with immunosuppression, further strengthening the immunosuppressive functions of M2 macrophages while concurrently suppressing CD8^+^ T cell activity, thus creating a microenvironment conducive to tumor growth and immune evasion. In our experiments, NDRG1 knockdown resulted in a significant reduction in LDHA expression, leading to impaired glycolytic activity, reduced lactate production, and decreased acidity in the TME. This metabolic alteration also significantly affected immune cell infiltration, with increased numbers of CD8^+^ T cells and decreased numbers of CD163^+^ M2 macrophages. These findings suggest that targeting NDRG1 could modulate the TME by reducing immunosuppressive signals and enhancing antitumor immunity. Furthermore, we investigated the therapeutic potential of NDRG1 as a target. The study showed that NDRG1 knockdown, in combination with immune checkpoint inhibitors such as PD‐1/PD‐L1 antibodies, significantly suppressed tumor growth and improved survival rates. This combination therapy not only increased CD8^+^ T cell infiltration but also reduced the number of M2 macrophages, further indicating that NDRG1‐targeted therapy could effectively enhance the efficacy of immune checkpoint blockade.

NDRG1 is a multifunctional intracellular protein that plays a critical role in various physiological and pathological processes, particularly in tumor biology.^[^ ^19^, ^20^, ^22^, ^23^ ^]^ It has been reported to have an oncogenic role in various cancers, underscoring its significance in cancer progression and metastasis.^[^ ^24^, ^25^, ^26^, ^27^, ^28^, ^29^ ^]^ The NDRG1 gene is located on the long arm of human chromosome 8 (8q24.22) and encodes a protein consisting of 394 amino acids, with its mRNA spanning ≈3.0 kilobase pairs.^[^ ^30^, ^31^ ^]^ NDRG1 is pivotal in the reprogramming of lactate metabolism, particularly in tumor and immune cells.^[^ ^32^ ^]^ Tumor cells alter their metabolic pathways to utilize lactate as an energy source, thereby supporting their proliferation and survival.^[^ ^18^ ^]^ NDRG1's regulatory role may further enhance this metabolic shift, promoting tumor growth and immune evasion. Additionally, NDRG1 is crucial in macrophage differentiation and function. Previous studies have shown that in NDRG1 knockout models, the differentiation of bone marrow cells into CD11b^+^ and F4/80^+^ macrophages is significantly impaired, highlighting the importance of NDRG1 in macrophage biology.^[^ ^17^ ^]^ Macrophages are key players in the immune system, responsible for phagocytosing pathogens and clearing dead cells, while also secreting immune‐regulatory cytokines and chemokines to modulate immune responses.^[^ ^33^ ^]^ To adapt to the metabolic demands of TME, macrophages undergo metabolic reprogramming, characterized by enhanced glycolysis and OXPHOS. M1‐type macrophages, associated with pro‐inflammatory responses, primarily rely on glycolysis and fatty acid synthesis (FAS) to support their functions, whereas M2‐type macrophages depend more on fatty acid oxidation (FAO) and OXPHOS to sustain their immunosuppressive and tumor‐promoting activities.^[^ ^34^, ^35^ ^]^ In the TME, the accumulation of lactate can drive macrophage polarization toward the M2 phenotype, which is typically associated with anti‐inflammatory responses, tissue repair, and tumor promotion.^[^ ^36^, ^37^ ^]^ M2 macrophages secrete anti‐inflammatory cytokines such as IL‐10 and TGF‐β, which contribute to creating an immunosuppressive environment that supports tumor growth and metastasis.^[^ ^38^ ^]^ The presence of NDRG1 further amplifies this metabolic reprogramming, promoting lactate accumulation in the TME and significantly enhancing the immunosuppressive functions of M2 macrophages. Notably, lactate influences macrophages not only through these mechanisms but also via a recently discovered post‐translational modification—lactylation—which plays an important role in macrophage function and TME modulation.^[^ ^39^ ^]^ This modification induces an open chromatin state, enhancing the transcription of genes associated with immunosuppression and driving macrophage polarization toward the M2 phenotype. The resulting M2 macrophages secrete immunosuppressive cytokines, which, in concert with elevated lactate levels in the TME, further impair CD8^+^ T cell function by inhibiting their cytotoxic activity and cytokine production. These combined effects contribute to the establishment of a TME that facilitates tumor immune evasion.^[^ ^40^ ^]^

There are still some limitations in our study that need to be acknowledged. Although we successfully demonstrated the role of NDRG1 in enhancing glycolysis and lactate production, the precise molecular mechanisms by which NDRG1 regulates these processes remain to be fully understood. Furthermore, our research primarily relied on in vitro and mouse models, which may not entirely reflect the complexity of human LUAD. This highlights the importance of clinical validation to confirm the therapeutic potential of targeting NDRG1 in human patients. Additionally, while scRNA‐seq and MR analysis were instrumental in identifying NDRG1, these methodologies come with inherent limitations, including potential biases in single‐cell data and the assumptions underlying MR analysis, particularly regarding pleiotropy. To enhance the robustness and validity of our findings, future research should consider incorporating additional genetic and epigenetic data.

In summary, our study provides a comprehensive exploration of the role of NDRG1 in metabolic reprogramming and immune regulation within the TME. We demonstrated that upregulation of NDRG1 enhances glycolysis and lactate production in tumor cells, driving the polarization of M2 macrophages and suppressing the activity of CD8^+^ T cells. These interactions contribute to the establishment of an immunosuppressive TME, thereby facilitating tumor progression and immune evasion. Targeting NDRG1, particularly in combination with immune checkpoint inhibitors, may offer a promising therapeutic approach for LUAD. These findings underscore the significance of NDRG1 as a potential therapeutic target and highlight the necessity for further research to fully elucidate its underlying mechanisms and therapeutic potential. Our study lays a solid foundation for future investigations into the role of NDRG1 in cancer biology and therapy.

## 4 Experimental Section

### Patients and Samples

The single‐cell sequencing data used in this study were obtained from two sources. First, 8 LUAD (lung adenocarcinoma) samples were collected using 10x Genomics single‐cell RNA sequencing technology from Zhongshan Hospital, Fudan University. Second, the GSE131907 dataset was downloaded from the GEO database (https://www.ncbi.nlm.nih.gov/geo/), which includes data from 11 LUAD patients and 11 normal lung tissue samples. RNA sequencing (RNA‐seq) transcriptome data (FPKM normalized) and clinical information for LUAD patients were downloaded from The Cancer Genome Atlas (TCGA) database, comprising data from 500 LUAD patients and 50 adjacent normal tissues.

To identify genetic factors associated with LUAD, genome‐wide association studies (GWAS) were conducted using data from the Transdisciplinary Research in Cancer of the Lung and The International Lung Cancer Consortium (TRICL‐ILCCO). The GWAS dataset included 85449 individuals, comprising 29863 LUAD cases and 55586 controls. Tumor and adjacent normal tissues were collected from 220 LUAD patients undergoing surgery at the Department of Thoracic Surgery, Zhongshan Hospital, Fudan University, between May 2021 and March 2023. All patients provided written informed consent, and the study was approved by the Institutional Review Board of Zhongshan Hospital (Approval No. B2021‐230R). Tissue samples were snap‐frozen in liquid nitrogen and stored at −80 °C until analysis.

### Preparation of Single‐Cell Suspensions

Tumor tissues were dissociated into single cells using mechanical and enzymatic methods with a Tumor Dissociation Kit (Miltenyi Biotec, Germany) according to the manufacturer's instructions. Dissociated samples were filtered through a 40 µm mesh and centrifuged at 300 × g for 7 min. Erythrocytes were removed using Red Blood Cell Lysis Solution (Sigma‐Aldrich), and dead cells were removed using a Dead Cell Removal Kit (Miltenyi Biotec), resulting in >90% cell viability.

### Differential Gene Expression Analysis

To identify key oncogenes in LUAD, single‐cell RNA sequencing (scRNA‐seq) data was first analyzed to classify cells into tumor and normal cells and identified differentially expressed genes between them. Subsequently, clinical genetic data was utilized from the International Lung Cancer Consortium (TRICL‐ILCCO) for eQTL (expression quantitative trait loci) and pQTL (cis‐protein quantitative trait loci) analyses. eQTL data were used to identify relationships between gene expression and genetic variations, while pQTL data were used to analyze the relationship between genetic variations and protein expression levels.

### Sensitivity and Pleiotropy Analyses

The inverse variance weighted (IVW) method was employed as the primary method and used weighted median, MR‐PRESSO, and MR‐Egger for sensitivity analyses. Through Cochran's Q test in heterogeneity analysis, the stability and reliability of the results was confirmed. In heterogeneity analysis, all SNPs corresponding to NDRG1 were evenly distributed, proving low heterogeneity and high homogeneity, making the results credible. Pleiotropy analysis also confirmed the stability and reliability of NDRG1 MR analysis results.

### Copy Number Variation (CNV) Analysis

To identify malignant epithelial cells, InferCNV to analyze CNVs in scRNA‐seq data was used. Raw count data were extracted from the Seurat object, using epithelial cells as reference to define the baseline. Epithelial cells were clustered with k‐means clustering based on CNV scores, defining clusters with high CNV scores as malignant. Further validation was performed by comparing with existing datasets and using additional CNV detection methods. Malignant cells were then annotated using the FindAllMarkers module to identify differentially expressed genes between malignant and non‐malignant epithelial cells.

### Cell Lines and Cell Culture

A549, H1299, PC9, H1975, LLC, HBE, and THP‐1 cell lines were used in this study. All cell lines were purchased from the Chinese Academy of Sciences Cell Bank. Cells were cultured in DMEM supplemented with 10% fetal bovine serum (FBS), 100 U ml^−1^ penicillin, and 100 U ml^−1^ streptomycin in a humidified incubator at 37 °C with 5% CO₂. THP‐1 cells were maintained in RPMI‐1640 medium supplemented with 10% FBS, 100 U ml^−1^ penicillin, and 100 U ml^−1^ streptomycin. For differentiation into macrophages, THP‐1 cells were treated with 100 ng mL^−1^ PMA for 24 h, followed by recovery in fresh RPMI‐1640 medium for 48 h.

### Quantitative Real‐Time PCR (qRT‐PCR)

qRT‐PCR was conducted to quantify mRNA expression levels of NDRG1, LDHA, and other key genes. Total RNA was extracted using TRIzol reagent (Invitrogen) following the manufacturer's protocol, and reverse transcription was performed using the PrimeScript RT Reagent Kit (Takara) with 1 µg of RNA. qRT‐PCR was carried out with SYBR Premix Ex Taq II (Takara) on a CFX96 Real‐Time PCR Detection System (Bio‐Rad). Primer sequences, including those for α‐tubulin (internal control), are listed in Table S1 (Supporting Information). The cycling conditions were: 95 °C for 2 min, followed by 40 cycles of 95 °C for 15 s, 60 °C for 30 s, and 72 °C for 30 s. Relative gene expression was calculated using the 2^(‐ΔΔCt) method. Results were expressed as mean ± SD from three independent experiments, and statistical significance was set at *p* < 0.05.

### Western Blotting (WB)

WB was performed to analyze the protein expression levels of NDRG1, LDHA, ubiquitinated proteins, and other relevant markers in LUAD tissues and cell lines. Tissue samples were homogenized in RIPA lysis buffer (Beyotime) containing protease and phosphatase inhibitors (Roche), and the lysates were centrifuged at 14000 × g for 15 min at 4 °C to collect the supernatants. LUAD cell lines were cultured, harvested, and lysed using RIPA buffer. Protein concentration was determined using the BCA Protein Assay Kit (Thermo Fisher Scientific). Equal amounts of protein (30–50 µg) were separated by SDS‐PAGE and transferred onto polyvinylidene difluoride (PVDF) membranes (Millipore). Membranes were blocked with 5% non‐fat dry milk in TBST (Tris‐buffered saline with 0.1% Tween 20) for 1 h at room temperature, and primary antibodies were incubated overnight at 4 °C. The primary antibodies used included anti‐NDRG1 (Abcam; ab124689; 1:1000), anti‐LDHA (Cell Signaling Technology; #3582; 1:1000), anti‐H3K18la (PTM BIO; PTM‐1406RM; 1:1000), anti‐Histone H3 (Cell Signaling Technology; #4499; 1:1000). Membranes were washed with TBST and incubated with HRP‐conjugated secondary antibodies (Cell Signaling Technology; 1:2000) for 1 h at room temperature. After the membranes were washed with Tris‐buffered saline‐Tween solution, the secondary antibodies were added to the membranes at room temperature. Finally, the protein bands were visualised with a BeyoECL Plus kit (Beyotime).

### Immunohistochemistry and Immunofluorescence Staining

Immunohistochemistry and immunofluorescence staining were performed to evaluate the expression of NDRG1 and related markers in tumor tissues. For immunohistochemistry, paraffin‐embedded sections were processed, including dewaxing, rehydration, antigen retrieval, and incubation with primary antibodies such as NDRG1 and CK7. Signals were developed using DAB and counterstained with hematoxylin. For immunofluorescence, fresh frozen sections were stained with primary antibodies targeting NDRG1, epithelial markers (e.g., CK7), and immune markers (e.g., CD8, CD163, CD274). Secondary antibodies conjugated with fluorophores were used, and nuclei were counterstained with DAPI. Staining results were analyzed using confocal microscopy to assess marker expression and their correlations.

### Extracellular Acidification Rate (ECAR) and Oxygen Consumption Rate (OCR) Analyses

ECAR and OCR were measured using a Seahorse XF Analyzer to assess glycolytic function and mitochondrial respiration in LUAD cells. Cells were seeded in Seahorse XF cell culture plates and allowed to adhere overnight. To measure ECAR, cells were treated with glucose, oligomycin, and 2‐deoxyglucose, detecting glycolytic flux via lactate production. For OCR measurements, cells were treated with oligomycin, FCCP, and rotenone/antimycin A, assessing mitochondrial respiration. This method enables real‐time assessment of metabolic activity, revealing basal respiration, ATP‐linked respiration, proton leak, and maximal respiratory capacity. The Seahorse XF Analyzer continuously measures oxygen concentration and proton flux, converting these into OCR and ECAR values. This dual‐parameter analysis provides comprehensive insights into how cells balance energy production between glycolysis and oxidative phosphorylation under different conditions, aiding in understanding the bioenergetic profiles of the cells.

### Protein‐Protein Interaction Network Analysis

A protein‐protein interaction (PPI) network was constructed using the STRING database to identify interactions between NDRG1 and other metabolic and regulatory proteins. The network included key proteins such as LDHA, MYCN, and HIF1A, and was visualized using Cytoscape software.

### Gas Chromatography‐Mass Spectrometry (GC‐MS) Analysis

GC‐MS was used to analyze metabolic intermediates in glycolysis and the TCA cycle in LUAD cells with knocked‐down NDRG1 expression. Cells were lysed, and metabolites were extracted and derivatized before analysis on a GC‐MS system. Quantitative analysis was performed to measure concentrations of citrate, isocitrate, α‐ketoglutarate, pyruvate, and lactate.

### Chromatin Immunoprecipitation Sequencing (ChIP‐seq) and ChIP‐qPCR

Macrophages were treated with lactate, then cross‐linked with formaldehyde, lysed, and sonicated to shear chromatin into fragments. Immunoprecipitation was carried out using an antibody specific to H3K18la. The bound chromatin was subjected to reverse cross‐linking, followed by DNA purification. Sequencing libraries were constructed from the immunoprecipitated DNA and sequenced using an Illumina platform to obtain genome‐wide profiles of H3K18la‐associated regions. For ChIP‐qPCR, the same immunoprecipitation procedure was followed. Selected genomic regions of interest, identified from sequencing data, were amplified using quantitative PCR with region‐specific primers. DNA enrichment levels at these loci were measured to assess the presence of H3K18la at specific sites.

### Mouse Models

C57BL/6 mice (4–6 weeks old) were purchased from the Model Animal Research Center of Nanjing University and housed in specific pathogen‐free conditions. LUAD mouse models were established by subcutaneous injection of 1 × 10⁶ LLC cells into the right flank of each mouse. Tumor volume was measured every 2 days using calipers, and calculated as (length × width^2^)/2. Experimental groups included LLC cells treated with shNDRG1 or shCtrl, with or without co‐injected macrophages. The animal experimental procedures were approved by the Animal Ethics Committee of Zhongshan Hospital, Fudan University (Shanghai, China), with Approval No. 2021–718.

### Bone Marrow‐Derived Macrophage (BMDM)

Bone marrow cells were isolated from the femurs and tibias of six‐week‐old female C57BL/6 mice and cultured in DMEM supplemented with 10% FBS and 20 ng mL^−1^ M‐CSF to differentiate into BMDMs. After 7 days of culture, BMDMs were harvested and treated with either PBS or lactate for 24 h prior to injection.

### Macrophage Depletion and Therapy

To study the role of macrophages in the tumor microenvironment, six‐week‐old female C57BL/6 mice were injected with 150 µL of clodronate liposomes via the tail vein to deplete macrophages. The efficiency of macrophage depletion was confirmed by flow cytometry analysis of peripheral blood mononuclear cells (PBMCs) and tissue macrophages. Bone marrow cells were isolated from donor mice and differentiated into BMDMs in vitro. During differentiation, BMDMs were treated with lactate to simulate tumor microenvironmental conditions. These lactate‐treated BMDMs were then injected into macrophage‐depleted tumor‐bearing mice to evaluate their effects on tumor growth and immune responses. This approach allowed for a controlled investigation of lactate's role in reprogramming macrophages and its impact on lung adenocarcinoma progression.

### Spatial Metabolomics Analysis

Spatial metabolomics was conducted to analyze metabolite distribution and concentration in LUAD and adjacent normal tissues. Freshly resected tissues were snap‐frozen, sectioned at 10 µm using a cryostat and mounted onto conductive glass slides for MALDI imaging mass spectrometry (IMS). Samples were prepared by spraying a solution of α‐cyano‐4‐hydroxycinnamic acid onto the sections, followed by MALDI‐IMS using an ultrafleXtreme MALDI TOF/TOF mass spectrometer. Mass spectra were acquired at a spatial resolution of 50 µm and processed with SCiLS Lab software to generate ion images for metabolites such as lactate, pyruvate, fumarate, α‐ketoglutarate, and succinate. Quantitative analysis compared metabolite intensities between tumor and normal tissues, defining regions of interest based on histological features. Validation of spatial metabolomics data was performed using LC‐MS, where tissues were homogenized in methanol, centrifuged, and the supernatant was dried and reconstituted for analysis. Metabolites were separated on a C18 column and quantified using external calibration curves, comparing levels between the tumor and adjacent normal tissues.

### Transwell Co‐Culture System

In the Transwell co‐culture system, A549 cells (LUAD cell line) were seeded in the lower chamber of a 24‐well Transwell plate (Corning, 3413), while THP‐1 derived macrophages were seeded in the upper chamber. This setup allows the cells to share the same medium but prevents direct cell‐to‐cell contact, facilitating paracrine signaling. A549 cells were treated with tumor‐conditioned medium (TCM) for 12 h prior to co‐culture to mimic the tumor microenvironment. Subsequently, the conditioned A549 cells were co‐cultured with THP‐1 macrophages for 12 h. After this period, the macrophages were transferred to a new Transwell insert and co‐cultured with CD8^+^ T cells for another 12 h to assess T cell activation and immune response. This method allowed for a controlled study of interactions between tumor cells, macrophages, and T cells, providing insights into the immunomodulatory roles of NDRG1 and lactate within the tumor microenvironment.

### T Cell Migration Assay

The T cell migration assay was conducted using a 24‐well Transwell system with 5.0 µm pore polycarbonate membrane inserts (Corning, 3421). Bone marrow‐derived macrophages (BMDMs) were co‐cultured with tumor cells or treated with tumor‐conditioned medium (TCM) for 12 h. CD8^+^ T cells were isolated from wild‐type mouse spleens using a CD8^+^ T Cell Isolation Kit (Miltenyi Biotec, 130‐095‐236), suspended in 200 µL of 1% FBS‐RPMI 1640 medium, and placed in the upper chamber with or without 100 nM SCH546738 (MedChemExpress, HY‐10017). The Transwell plates were incubated at 37 °C in a 5% CO₂ atmosphere for 4 h. CD8^+^ T cell migration from the upper to the lower chamber was assessed by collecting and counting cells in the lower chamber. Additionally, primary CD8^+^ T cells were isolated from human peripheral blood using the EasySep Human CD8^+^ T Cell Enrichment Kit (STEMCELL Technologies, 17 953). These T cells were placed in the upper chamber, while primary human macrophages stimulated with LUAD‐TCM were placed in the lower chamber. CD8^+^ T cell migration was evaluated after 6 h of incubation at 37 °C, 5% CO₂ by counting cells in the lower chamber.

### 2‐NBDG Glucose Uptake Assay

Cells were cultured in a glucose‐free medium (Thermo Fisher, USA) for 48 h prior to the assay. For glucose uptake measurement, cells were incubated in 100 µL glucose‐free medium containing 200 µM 2‐NBDG (MKBio, China) at 37 °C for 80 min in the dark to protect from photo‐degradation. After incubation, cells were washed twice with cold 1 × PBS to remove excess 2‐NBDG. Fluorescence intensity of 2‐NBDG uptake was quantified under a fluorescence microscope equipped with a FITC filter. The experiment was independently repeated three times to ensure reproducibility and consistency of results.

### Flow Cytometry

Dead cells were excluded using Fixable Viability Dye eFluor 450 (Invitrogen), which was applied during the initial steps of sample preparation, prior to antibody staining. Surface staining was performed at 4 °C for 30 min in the dark using the following fluorochrome‐conjugated antibodies: CD45‐APC (clone 30‐F11, BD Biosciences) for total leukocytes; CD11b‐PerCP‐Cy5.5 (clone M1/70, BD Biosciences) for myeloid cells; Gr1‐PE‐Cy7 (clone RB6‐8C5, BD Biosciences) for myeloid‐derived suppressor cells (MDSCs); F4/80‐BV650 (clone BM8, BioLegend) for macrophages; CD86‐BV711 (clone 24F, BD Biosciences) for M1‐like macrophages; CD163‐BV605 (clone S15049I, BioLegend) for M2‐like macrophages; CD3‐BV510 (clone 145‐2C11, BD Biosciences) for T cells; CD4‐FITC (clone RM4‐5, BD Biosciences) for CD4⁺ T cells; and CD8‐APC‐Cy7 (clone 53–6.7, BD Biosciences) for CD8⁺ T cells.  After surface staining, cells were washed and immediately acquired. Data acquisition was performed on a BD LSRFortessa flow cytometer (BD Biosciences), and data were analyzed using FlowJo software v10 (Tree Star Inc.). The gating strategy included sequential exclusion of debris based on forward and side scatter (FSC versus SSC), followed by doublet discrimination using FSC‐A versus FSC‐H. Viable single cells were defined as eFluor 450‐negative. Total immune cells were identified as CD45⁺, and within this population, myeloid cells were gated as CD11b⁺. These were further stratified into Gr1⁺ MDSCs and F4/80⁺ macrophages. Macrophage subsets were delineated by CD86 and CD163 expression, corresponding to M1‐like and M2‐like phenotypes, respectively. T cells were defined as CD3⁺ and further classified into CD4⁺ and CD8⁺ subsets.

### Statistical Analysis

Statistical analyses were performed using GraphPad Prism version 8.0 and R software. Survival analysis was conducted using the Kaplan–Meier method and differences between survival curves were assessed using the log‐rank test. A multivariate Cox proportional hazard regression model was applied to identify independent prognostic factors. The data shown (unless otherwise indicated) represent results from at least three independent experiments. Data are expressed as the mean ± standard deviation (SD) or standard error of the mean (SEM). Pearson correlation analysis was used to assess relationships between variables. Statistical significance between two groups was determined using two‐sided Student's t‐test or Wilcoxon rank‐sum test, depending on the data distribution. P‐value < 0.05 was considered statistically significant.

## Conflict of Interest

The authors declare no conflict of interest.

## Author Contributions

G.W., H.C., J.Y., and Y.Z. contributed equally to this work. G.W., Y.H., Y.Z., B.P., H.S., M.L., and M.Z. contributed to the design of experiments and preparation of the article. G.W., H.C., J.Y., Y.Z., Y.H., J.L., Y.B., and G.S. performed animal studies. Y.H., H.C., G.W., J.Y., Y.Z., H.S., Y.Z., B.P., and G.B. contributed to the acquisition of the data. G.W., Y.H., H.C., and J.Y. contributed to the data analyze and writing of the manuscript. Y.H., L.W., and W.G. made significant revisions to the manuscript and ultimately approved it for publication. All authors commented on the manuscript.

## Supporting information



Supporting Information

Supplemental Table 1

## Data Availability

The data that support the findings of this study are available from the corresponding author upon reasonable request.
