# Supplementary material for: NDRG1‐Driven Lactate Accumulation Promotes Lung Adenocarcinoma Progression Through the Induction of an Immunosuppressive Microenvironment
Source: Adv Sci (Weinh). 2025 Jun 20;12(33):e01238. doi: 10.1002/advs.202501238 (PMC12412559; doi:10.1002/advs.202501238)
Supplement: Supplementary file 1 — Supporting Information [file ADVS-12-e01238-s001.docx]

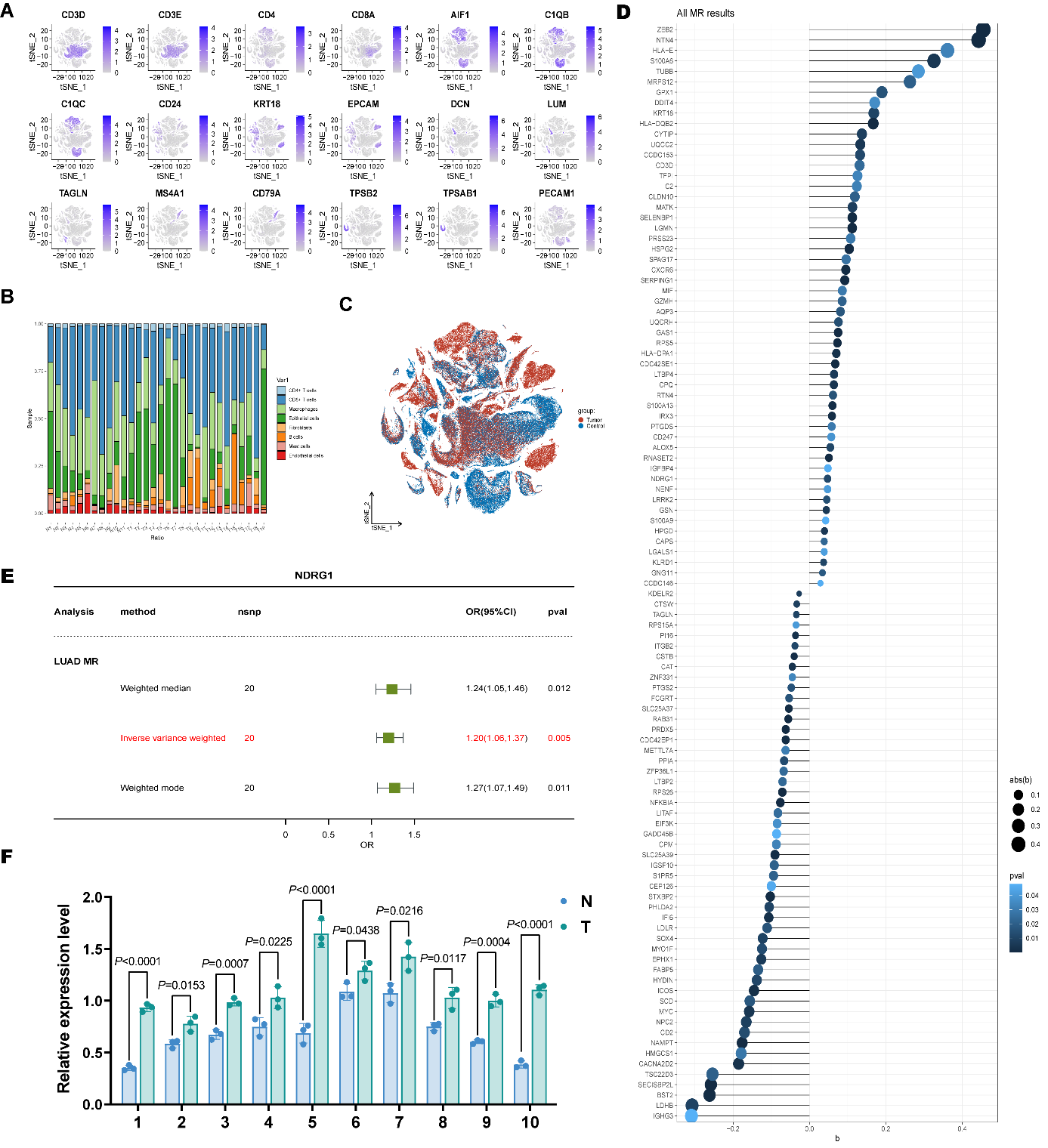
Figure S1: Comprehensive analysis of cell types and gene expression in LUAD

(A) Clusters of quality-controlled single cells based on the expression of typical marker genes. (B) Cell proportion plot. (C) t-SNE plot of normal and tumor cells. (D) eQTL analysis results. (E) Forest plot for the causality of NDRG1 in LUAD derived from MR analysis. (F) Western blot analysis of NDRG1 in 10 pairs of cancer and adjacent non-tumor samples **(n=3)**. **Differences were considered statistically significant at** p **< 0.05.**


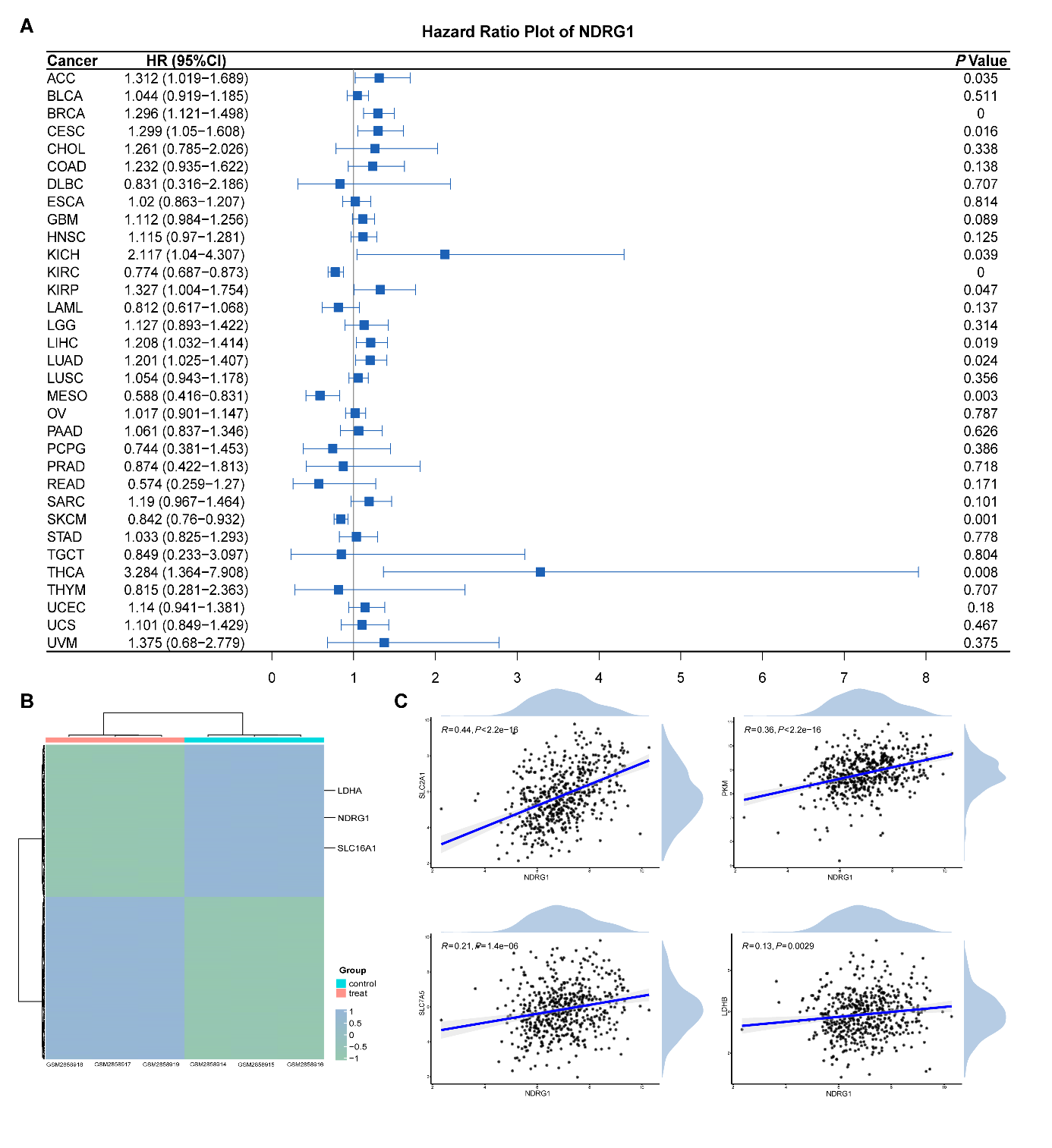
Figure S2：Analysis of NDRG1 in various cancers and its correlation with key metabolic genes

1. Hazard ratio plot of NDRG1 across various cancer types. (B) Heatmap showing the expression levels of NDRG1, LDHA, and SLC16A1 in control and treatment groups. (C) Correlation plots showing the relationship between NDRG1 and key metabolic genes (SLC16A1, PKM, HK1, and LDHB).


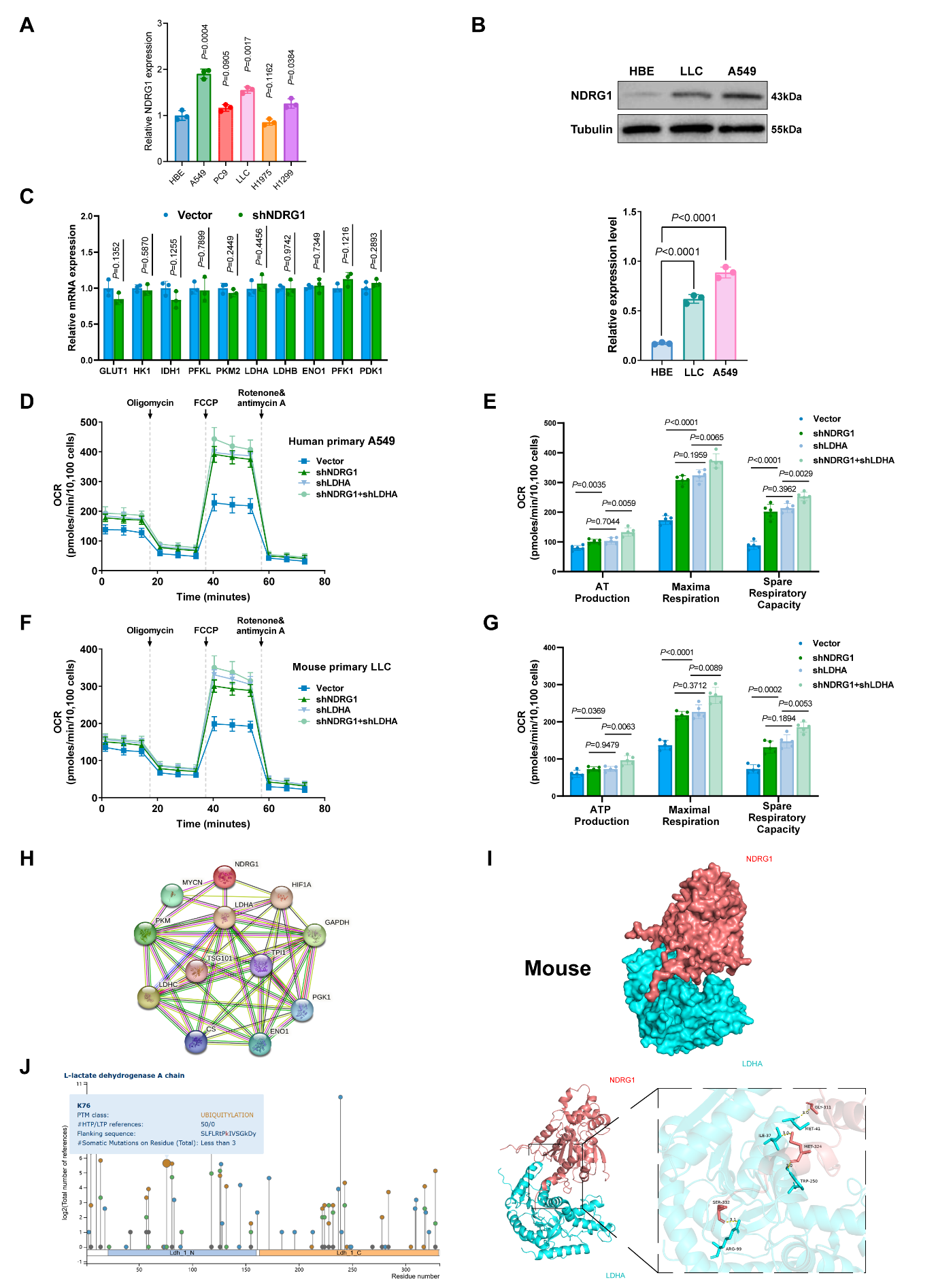


Figure S3: NDRG1 and LDHA cooperation in regulating cellular metabolism.

1. Relative NDRG1 expression levels in different lung cancer cell lines (HBE, A549, PC9, LLC, H1975, H1299) **(n=3)**. (B) Western blot analysis of NDRG1 levels in A549 and LLC cells **(n=3)**. (C) mRNA levels of metabolic genes (GLUT1, HK1, IDH1, PFKL, PKM2, LDHA, LDHB, ENO1, PYK1, PDK1) under different NDRG1 expression conditions **(n=3)**. (D, E) OCR response curve and quantitative analysis (ATP Production, Maximal Respiration, Spare Respiratory Capacity) of A549 cells under different conditions (Vector, shNDRG1, shLDHA, shNDRG1+shLDHA) after treatment with oligomycin, FCCP, and rotenone & antimycin A **(n=5)**. (F, G) OCR response curve and quantitative analysis (ATP Production, Maximal Respiration, Spare Respiratory Capacity) of LLC cells under different conditions (Vector, shNDRG1, shLDHA, shNDRG1+shLDHA) after treatment with oligomycin, FCCP, and rotenone & antimycin A **(n=5)**. (H)Protein-Protein Interaction (PPI) network analysis demonstrating connections between NDRG1, LDHA, and other metabolic-related genes. (I) Molecular docking analysis showing the complex between mouse NDRG1 and LDHA, with a focus on interaction sites. (J) Analysis Results of LDHA Ubiquitination Sites. **Differences were considered statistically significant at** p **< 0.05.**


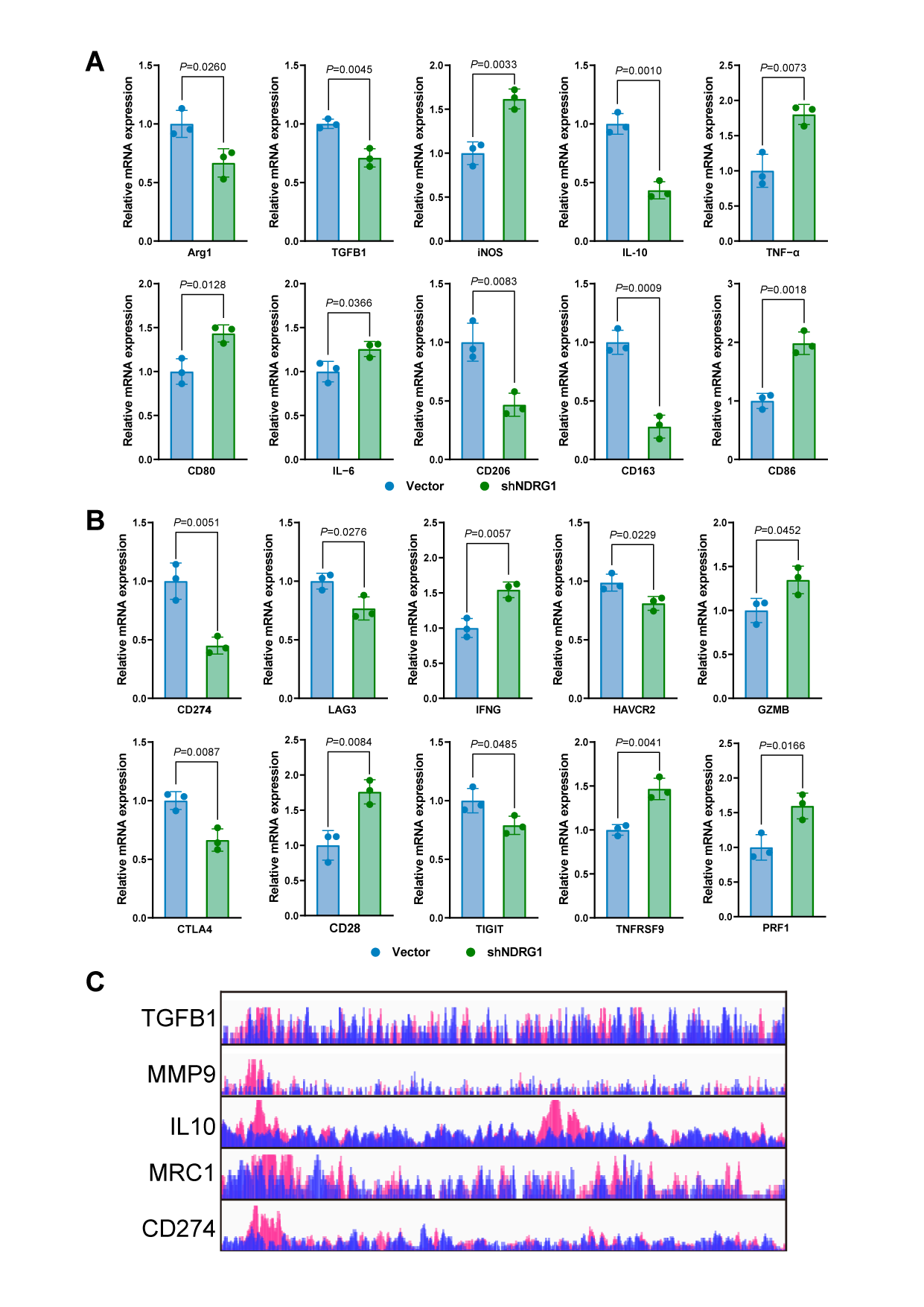
Figure S4: Impact of NDRG1 knockdown on immune markers and H3K18la enrichment.

1. qRT-PCR results showing relative mRNA expression levels of M2 macrophage markers (CD163, CD206, Arg1, IL-10) and M1 macrophage markers (CD80, CD86, iNOS) in THP-1 macrophages after co-culture with NDRG1 knockdown (shNDRG1) and control (Vector) A549 cells **(n=3)**. (B) qRT-PCR results showing relative mRNA expression levels of T cell activation markers (SELL, CCR7), cytotoxic effectors (GZMA, PRF1), and immune checkpoint genes (PD-L1, CTLA4) in CD8^+^ T cells after co-culture with THP-1 macrophages **(n=3)**. (C) ChIP-seq analysis showing H3K18la enrichment at the gene promoter regions of TGFB1, MMP9, IL10, MRC1, and PD-L1. **Differences were considered statistically significant at** p **< 0.05.**

**Supplementary Table 1： Sequences for shRNA used in this study**
